# Cytotoxic Effect of Escitalopram/Etoposide Combination on Etoposide-Resistant Lung Cancer

**DOI:** 10.3390/ph18040531

**Published:** 2025-04-05

**Authors:** Serap Özkaya Gül, Beyzanur Şimşek, Fidan Yıldız, Esra Aydemir

**Affiliations:** Department of Biology, Faculty of Science, Akdeniz University, Antalya TR-07058, Turkey; 202151006001@ogr.akdeniz.edu.tr (S.Ö.G.); 202351006002@ogr.akdeniz.edu.tr (B.Ş.); 202351005006@ogr.akdeniz.edu.tr (F.Y.)

**Keywords:** escitalopram oxalate, etoposide, etoposide-resistant lung cancer

## Abstract

**Background:** Antidepressants are a class of pharmaceuticals utilized for the management of many psychiatric disorders, including depression. A considerable number of antidepressants, particularly selective serotonin reuptake inhibitors (SSRIs), have been documented to demonstrate significant anticancer properties in various cancer cell lines. **Objectives:** The aim of this study was to evaluate the selective cytotoxic and apoptotic effects of escitalopram oxalate (ES) alone and in combination with etoposide (ET) on ET-resistant A549 (A549/90E) lung cancer cells. **Methods:** The cytotoxic effects of the drugs were determined by CCK-8, trypan blue, and neutral red assays. Apoptosis was observed by Annexin V fluorescein isothiocyanate (FITC)/PI and mitochondrial membrane potential (ΔΨm) assays. Moreover, the effects of the drugs, alone and in combination, on apoptosis-related proteins, caspase-3, PTEN, and resistance-related P-gP were determined by ELISA. The relationship between drugs and lung cancer was determined with protein–protein interaction (PPI) network analysis. **Results:** Our results revealed that ES significantly exerted cytotoxic effects on both wild-type and A549/90E cells compared with BEAS-2B cells. The IC_50_ values of 48.67 and 51.6 μg/mL obtained for ET and ES, respectively, at the end of 24 h of incubation for A549 cells were applied reciprocally for each cell by including BEAS-2B together with the 2xIC_50_ and ½ IC_50_ values. The results of each combination were statistically evaluated with combination indices (CIs) obtained using the Compusyn synergistic effect analysis program. Combination doses with a synergistic effect in A549 and A549/90E cells and an antagonistic effect in BEAS-2B cells have been determined as ½ IC_50_ for ET and ½ IC_50_ for ES. ET ½ IC_50_, ES ½ IC_50_, and an ET ½ IC_50_ + ES ½ IC_50_ combination caused 18.37%, 55.19%, and 57.55% death in A549 cells, whereas they caused 44.9%, 22.4%, and 51.94% death in A549/90E cells, respectively. In A549 cells, the combination of ES ½ IC_50_ and ET ½ IC_50_ caused increased levels of caspase-3 (*p* < 0.01) and P-gP (*p* < 0.001), while PTEN levels remained unchanged. The combination resulted in an increase in caspase-3 (*p* < 0.001) and PTEN (*p* < 0.001) amounts, alongside a decrease in P-gP (*p* < 0.01) levels in A549/90E cells. The death mechanism induced by the combination was found to be apoptotic by Annexin V-FITC and ΔΨm assays. **Conclusions:** Based on our findings, ES was observed to induce cytotoxic and apoptotic activities in A549/90E cells in vitro. ES in combination therapy is considered to be effective to overcome ET resistance by reducing the amount of P-gP in A549/90E cells.

## 1. Introduction

Depression is a severe mood disorder that has profound effects on an individual’s emotional, cognitive, and social functioning. Characterized by a persistent sense of sadness, profound hopelessness, an inability to derive pleasure from life, and feelings of emptiness, this condition may lead to alterations in sleep patterns and eating habits, difficulties in concentration, and, in extreme cases, suicidal ideation. Depression adversely affects an individual’s mood and daily activities, often manifesting through symptoms such as psychomotor dysfunction. This disorder is shaped by the interplay of biological and psychosocial factors, representing a complex phenomenon with significant implications at both individual and societal levels [1,2].

**Figure 1:**
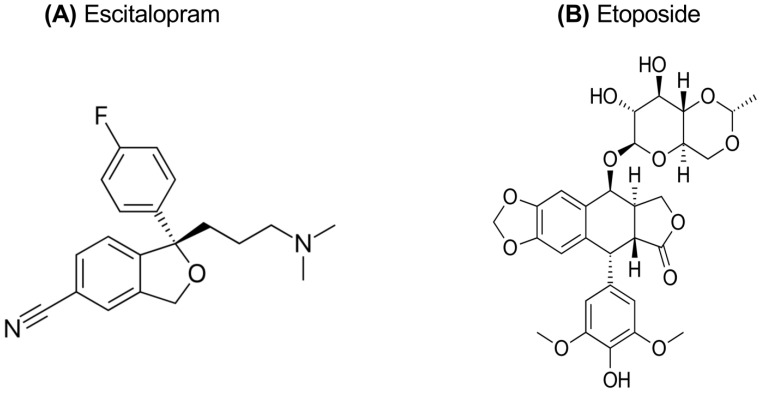
Chemical structure of escitalopram oxalate (**A**) and etoposide (**B**).

The treatment of depression is a multifaceted process involving a combination of pharmacological and non-pharmacological approaches. Antidepressants used in pharmacotherapy are classes based on their different mechanisms of action. These include selective serotonin reuptake inhibitors (SSRIs), serotonin–norepinephrine reuptake inhibitors (SNRIs), tricyclic antidepressants (TCAs), norepinephrine reuptake inhibitors (NARIs), and norepinephrine–dopamine reuptake inhibitors (NDRIs) [1]. Antidepressants are generally classified into three main groups: first-generation, second-generation, and third-generation. First-generation antidepressants include monoamine oxidase inhibitors (MAOIs) and tricyclic antidepressants, while second-generation antidepressants include SSRIs, SNRIs, norepinephrine and specific reuptake inhibitors (NaSSAs), serotonin antagonist/reuptake inhibitors (SARIs), and melatonergic agents. These treatments aim to alleviate depression symptoms and enhance an individual’s quality of life [3,4]. SSRIs and SNRIs are frequently preferred in the treatment of depression due to their high safety profiles and tolerability levels. Conversely, MAOIs and TCAs are less commonly utilized in contemporary practice, as their safety profiles are comparatively lower than those of SSRIs and SNRIs [5]. In a systematic review and meta-analysis conducted by Cipriani et al., agomelatine, escitalopram, mirtazapine, paroxetine, venlafaxine, and vortioxetine were found to demonstrate superior efficacy compared to other antidepressants [6]. By contrast, fluoxetine, fluvoxamine, reboxetine, and trazodone were identified as having inferior efficacy. Fluoxetine, fluvoxamine, paroxetine, sertraline, citalopram, and escitalopram from the SSRI group are generally regarded as first-line drugs in the treatment of depression [1]. Escitalopram oxalate (ES), commercially known under the brand names Cipralex and Lexapro, is an antidepressant classified as an SSRI and represents the active enantiomer of citalopram [7]. The (S)-enantiomer form of citalopram, ES, is known as [(S)-(+)-1-[3-(dimethyl-amino) propyl]-1-(p-fluorophenyl)-5-phthalancarbonitrile oxalate], and the chemical structure of ES is shown in Figure 1A [8].The U.S. Food and Drug Administration (FDA) has approved the use of ES for the treatment of both the acute and maintenance phases of major depressive disorder in adults and adolescents aged 12 to 17 years. The drug’s high selectivity and strong binding affinity to the serotonin transporter (SERT) make it effective in treating a variety of psychiatric disorders, including major depression and anxiety disorders. ES facilitates the inactivation of SERT, leading to increased levels of serotonin in the synaptic cleft, thereby enhancing its therapeutic effects. ES is mainly processed in the liver by certain enzymes known as cytochrome P-450 isoenzymes, specifically 2C19, 3A4, and 2D6 [9,10].

Cancer is currently the second leading cause of death, following cardiovascular diseases, worldwide [11]. Lung cancer remains the most prevalent cause of cancer-related mortality, accounting for more than one million deaths among both men and women. Following lung cancer, the cancers associated with the highest mortality rates are colorectal, liver, breast, and gastric cancers. In terms of gender, men are observed to have a higher risk of cancer-related death compared to women. Among men, lung cancer remains the leading cause of cancer-related death, followed by liver, colorectal (colon and rectum), and gastric cancers. In women, the incidence of lung and breast cancers is almost equal, with breast cancer being the most prevalent [12]. Lung cancer is generally classified into two main groups: small cell lung cancer (SCLC) and non-small cell lung cancer (NSCLC) [13]. Compared to NSCLC, small cell lung cancer is more aggressive and invasive, often metastasizing to distant organs such as the brain, skeletal system, and lymphatic tissues. Non-small cell lung cancer is divided into three main subtypes: adenocarcinoma, squamous cell carcinoma, and large cell carcinoma. Among these subgroups, large cell carcinomas exhibit rapid growth and a high potential for metastasis, while adenocarcinomas comprise approximately 40% of non-small cell lung cancer [14,15]. In cancer treatment, different approaches are adopted depending on the specific type and stage of cancer. Traditional treatment modalities comprise surgical intervention, chemotherapy, and radiotherapy, whereas modern therapeutic strategies incorporate hormone therapy, immunotherapy, stem cell therapy, and dendritic cell-based immunotherapy. These treatment methods can be applied either individually or in combination to achieve synergistic effects. The success of cancer therapy is highly dependent on the appropriate selection of treatment modalities and their adaptation to the patient’s health status [16,17,18].

Etoposide (ET) is a topoisomerase II inhibitor belonging to the class of podophyllotoxin derivatives. Podophyllotoxin, a lignan compound naturally obtained from the Podophyllum species, is a semi-synthetic derivative and is among the chemotherapeutic agents used in cancer treatment [19]. The chemical structure of etoposide is known as [(9-[(4,6-O-ethylidene-β-D-glucopyranosyl) oxy]-5,8,8a,9-tetrahydro-5-(4-hydroxy-3,5-dimethoxyphenyl) furo [3′,4′:6,7] naphtho [2,3-d]-1,3-dioxol6(5aH)-one)] and shown in Figure 1B. ET is a potent chemotherapeutic agent that has been effective in the treatment of various malignancies, including lung cancer, lymphoma, leukemia, testicular cancer, ovarian cancer, and sarcoma [20]. However, like most anti-neoplastic drugs, ET has limited efficacy alone and is usually used in combination therapies. ET is recognized as a topoisomerase II (TOP2) inhibitor, and this inhibition forms the principal mechanism underlying the drug’s anticancer activity. By inhibiting the topoisomerase II enzyme, ET blocks DNA replication and transcription, thereby triggering the process of programmed cell death (apoptosis) in the cell. These properties make ET an effective treatment option for various cancer types [20,21].

Although chemotherapy is currently regarded as an essential option in cancer treatment, drug resistance poses a significant challenge that severely limits therapeutic success. Approximately 90% of chemotherapy failures occur in the invasion and metastatic processes due to the drug resistance of cancer cells [22]. Many cancer types that are initially sensitive to chemotherapy may develop resistance over time due to factors such as DNA mutations, metabolic changes, and drug inhibition mechanisms. Drug resistance reduces the therapeutic effect of a drug, making disease treatment difficult and adversely affecting overall survival. Multidrug resistance (MDR) is defined as the development of resistance by cancer cells to different chemotherapeutic agents, and the ATP-binding cassette (ABC) transporter superfamily, such as P-glycoprotein (P-gP: ABCB1\/MDR1), ABCG2 (breast cancer resistance protein: (BCRP); ABCG2), and multidrug resistance-associated protein 1 (MRP1, (ABCC1)), are involved in this resistance. Combination therapy is considered to be one of the most effective methods to overcome such resistance [23,24]. P-gP, as an ATP-dependent transporter, plays a critical role in the development of drug resistance in cancer cells. P-gP was first identified in Chinese hamster ovary cells in 1974. This 170 kDa membrane glycoprotein, encoded by the *MDR1* gene, contains two homologous structures, each consisting of six transmembrane domains (TMDs) and one nucleotide-binding domain (NBD). Using energy derived from ATP hydrolysis, P-gP actively transports anticancer drugs out of the cell against a concentration gradient, thereby reducing the intracellular accumulation of chemotherapeutic agents [21,25]. P-gP has a wide range of chemotherapeutic substrates, such as taxanes, vinca alkaloids, epipodophyllotoxins, anthracyclines, and actinomycin D [24]. The active transport of these drugs out of tumor cells reduces the cytotoxic effect of chemotherapy and limits the success of therapy. The overexpression of P-gP in tumor cells leads to drug resistance by maintaining intracellular drug concentrations below the threshold of therapeutic efficacy. P-gP also plays a crucial role in the absorption and excretion of drugs in organs such as the intestine, liver, kidney, and brain under physiological conditions. It is expressed at high levels especially in the blood–brain barrier and apical membrane of epithelial cells [26,27]. The effect of P-gP on drug resistance presents a significant challenge in cancer chemotherapy. Both traditional cytotoxic agents and novel, targeted anticancer drugs are susceptible to efflux via P-gP. Therefore, the development of P-gP inhibitors is critical for overcoming drug resistance and enhancing the efficacy of chemotherapy [21,28].

Apoptosis is an evolutionarily conserved and tightly regulated cell death program that plays a critical role in various normal physiological processes, ranging from embryogenesis to adult tissue homeostasis. Apoptosis can be induced by a variety of stimuli, including low-dose radiation, hypoxia, heat, and cytotoxic drugs. Treatments such as radiotherapy and chemotherapy can induce apoptosis by causing irreparable DNA damage in cells, which is recognized as an important mechanism in the efficacy of treatment [29,30]. Apoptosis occurs through two basic regulated pathways in response to intracellular and extracellular signals: the intrinsic (mitochondrial) pathway and the extrinsic (death receptor) pathway. The intrinsic pathway is initiated by cellular stress signals such as ultraviolet radiation, hypoxia, DNA damage, or growth factors, whereas the extrinsic pathway is initiated by ligands binding to death receptors such as FAS, TNF, and TRAIL. Both pathways utilize cysteine proteases known as caspases to initiate and regulate the apoptotic process. Caspases are categorized into three main groups based on their roles at different stages of apoptosis: initiator, effector, and inflammatory caspases. Initiator caspases (caspase-2, -8, -9, and -10) are activated at the onset of apoptotic signaling and enable the activation of effector caspases (caspase-3, -6, -7) [30,31]. The *CASP3* gene encodes the protein caspase-3, which belongs to the endoprotease (cysteine-aspartic protease) family. It is initially synthesized in an immature pro-caspase form, and its activation occurs through specific proteolysis. Caspase-3, which can be activated through both the extrinsic (death receptor-mediated) and intrinsic (mitochondrial) pathways, serves as a critical regulator of the apoptotic process. In addition to its central role in apoptotic cell death mechanisms, caspase-3 also involved in non-apoptotic processes such as tissue differentiation, regeneration, and neural development [32].

The tumor suppressor gene *p53* is the most frequently mutated gene and is considered one of the most common genetic alterations in cancer. Mutations in *p53* result in the loss of its tumor-suppressive properties and can also promote tumor progression. p53 functions as a transcription factor that regulates cellular processes, including cell cycle control, apoptosis, autophagy, and metabolism. Under conditions of cellular stress, it supports the repair process by arresting the cell cycle to prevent the proliferation of cells with damaged or mutated DNA or triggers apoptosis to eliminate these cells. This process forms the basis of the tumor suppressor function of p53. Furthermore, p53 activates both intrinsic and extrinsic apoptotic pathways by regulating the transcription of proteins such as PUMA, Bid, Bax, TRAILR2, and CD95. Through these mechanisms, p53 plays a critical role in inhibiting tumor development and maintaining of cellular homeostasis [33,34]. Phosphatase and tensin homolog (*PTEN*) is a critical tumor suppressor gene that plays essential roles in suppressing cell growth and increasing sensitivity to apoptosis. *PTEN*, one of the most frequently mutated genes in cancer, regulates essential cellular functions such as cell proliferation, growth, survival, and metabolism as a negative regulator of the PI3K/AKT/mTOR signaling pathway. The loss of *PTEN* function is considered a critical genetic event in tumorigenesis and progression. Moreover, *PTEN* plays a key role in cancer prevention by regulating various cellular processes such as angiogenesis, the cell cycle, apoptosis, cell migration, and invasion [35,36,37]. *PTEN* and *p53* are the two most frequently mutated or inactivated tumor suppressor genes in human cancers. Functional interactions between *PTEN* and *p53* are available at both the transcriptional and protein levels. In response to cellular stresses such as DNA damage, p53 binds to a specific region of the *PTEN* promoter, thereby enhancing its expression. Also, *PTEN* indirectly regulates p53 levels by modulating the transcription of MDM2, a critical regulator of p53, and plays an essential role in p53-mediated apoptosis. Additionally, *PTEN* expression is upregulated by transcription factors such as p53, PPARγ, and EGR1, while it is negatively regulated by molecules such as TGF-β, NF-κB, and Jun. This bidirectional interaction between *PTEN* and *p53* serves as a critical control mechanism to maintain the balance between cellular survival and death, and the disruption of these interactions can initiate tumor formation and progression [36,38,39]. Therefore, the mechanism of apoptosis involves crosstalk between intrinsic and extrinsic pathways, adding to the complexity of the regulatory mechanisms of apoptosis. The disruption of these pathways is associated with abnormal cell development and disease. Thus, apoptosis is recognized as a fundamental mechanism in maintaining cellular homeostasis and organismal health [40].

There are several strategies for cancer treatment, often involving a combination of multiple therapies [41]. Network pharmacology integrates systems biology, pharmacology, and computational analysis technology to explore complex interactions among components, diseases, and targets [42]. Network pharmacology is a methodical strategy to elucidate the dynamic relationships among drugs, possible targets, and related pathways through the construction of drug–target–disease interactions [43]. Bioinformatics studies provides a robust framework for examining complex mechanisms [44,45]. Escitalopram oxalate is better tolerated and less toxic than other SSRIs, including citalopram, paroxetine, fluoxetine, and sertraline. In addition, little is known about the effects of escitalopram oxalate on drug resistance [15]. Compared to SSRIs antidepressants, escitalopram and its metabolites are weak inhibitors of CYP2D6 and have been reported not to cause significant inhibition of other CYP1A2, CYP2C9, CYP2C19, and CYP3A4 isoforms [46,47,48]. Considering drug interactions, escitalopram is more tolerable than other SSRI-group drugs and exhibits weaker/insignificant side effects [48]. There are many studies indicating that antidepressant drugs exhibit cytotoxic effects on cancer cells and increase sensitivity to chemotherapeutic drugs in combination applications [49,50,51]. Pharmacokinetic interactions can be seen between antidepressants and anticancer drugs. Therefore, it is important to consider these interactions when selecting antidepressant and anticancer drugs [52]. It has been reported that antidepressants can be used in combination with chemotherapeutic drugs at tolerable doses and can be considered as chemosensitizer agents [53]. Due to drug resistance, etoposide has been the subject of more combination studies in recent years [54,55,56]. This study aims to investigate the cytotoxic effects of ES, ET, and their combinations on human lung cancer cells, including both wild-type and ET-resistant variants, in vitro by using WST-1, Annexin-V, ΔΨm assays, and a network pharmacology approach to predict target genes.

## 2. Results

### 2.1. Cytotoxic Effects of ES and ET

The cytotoxic effects of ES and ET on A549, A549/90E, and BEAS-2B cell lines were evaluated using the CCK-8 test, which identifies live cells based on mitochondrial dehydrogenase activity. Cells were treated with drugs in the range of 5 to 500 μg/mL for 24, 48, and 72 h of incubation.

**Figure 2:**
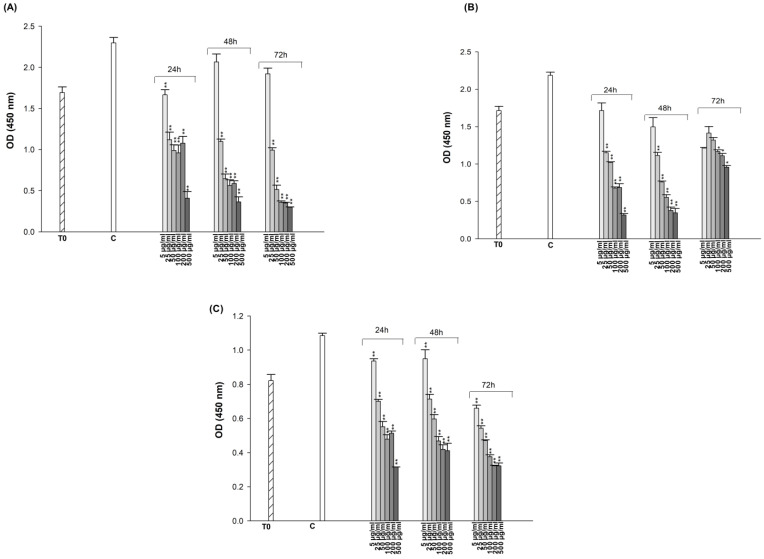
Cytotoxic effect of ET on A549 (**A**), A549/90E (**B**), and BEAS-2B (**C**) cells after 24, 48, and 72 h of incubation. The statistical analysis of the data was carried out by Student *t*-test. * *p* < 0.05, and ** *p* < 0.01 were considered to indicate a statistically significant differences compared to control group.

**Figure 3:**
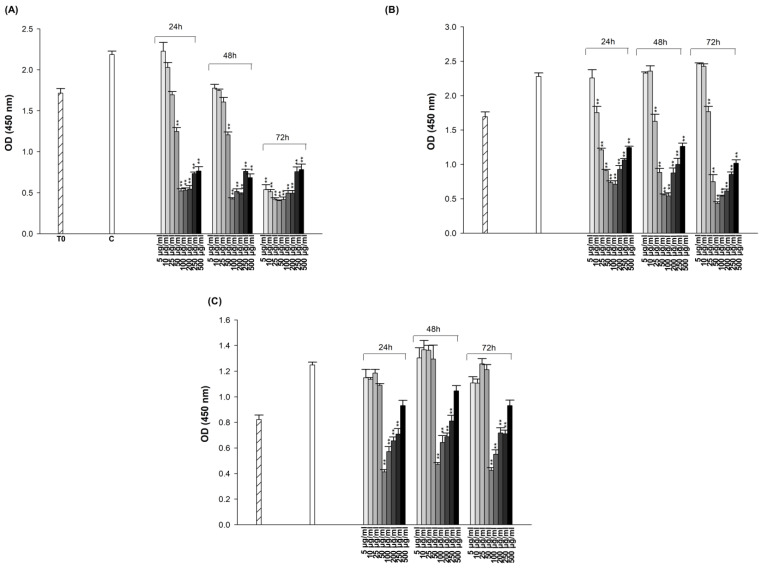
Cytotoxic effect of ES on A549 (**A**), A549/90E (**B**), and BEAS-2B (**C**) cells after 24, 48, and 72 h of incubation. The statistical analysis of the data was carried out by Student *t*-test. ** *p* < 0.01 were considered to indicate a statistically significant differences compared to control group.

The OD values from the CCK-8 assay were analyzed with Graph-Pad Prism version 4.00 (Graph-Pad Software, San Diego, CA, USA), plotted separately for each cell line with SigmaPlot 10 (Figure 2 and Figure 3). The obtained data clearly indicate that both ES and ET exhibit different cytotoxic effects in each cell at the doses and incubation times we tested. As shown in Figure 3, the cytotoxicity data demonstrated that higher concentrations of ES (from 100 to 500 μg/mL) appear to lead to increased cell survival. There seems to be inconsistency between in vitro and in vivo results in the literature regarding the effects of antidepressants on tumor growth. The effect of an antidepressant on cancer prognosis parameters appears to depend on several factors: (1) the interaction of the specific SSRI with tumor cells, (2) the dose used and its potential cytotoxicity, and (3) the specific cancer cell type [57]. ES inhibits the function of SERT via binding to the orthosteric binding site where the substrate 5-HT binds. The inhibition of SERT leads to elevated 5-HT levels at the synapses, enhancing serotonergic neurotransmission, which is believed to underlie the antidepressant effects of these medications. One or more allosteric sites may exist on SERT in addition to the orthosteric binding site. ES that binds to the allosteric site can modulate the properties of the orthosteric binding site and potentially affect the physical interaction between SERT and its interacting proteins, thus modulating SERT by associated proteins [58]. The use of SSRI antidepressants has the general effect of increasing 5-HT levels in the synaptic cleft and plasma by blocking 5-HT reuptake [59]. The increase in 5-HT levels can trigger cell proliferation at high concentrations [60,61,62]. The obtained CCK-8 test results have also been validated with trypan blue (Table S1) and neutral red tests (Figures S1 and S2). Concentrations that reduced cell viability by 50% (IC_50_), if detectable in the dose range tested, are presented in Table 1. The IC_50_ values that could be determined within the tested dose range for both drugs were obtained at the end of 24 h in A549 cells, at the end of 72 h in A549/90 E cells, and at the end of both 24 h and 72 h in BEAS-2B cells. Since the IC_50_ values obtained in A549 cells were lower than those obtained in BEAS-2B cells after both 24 and 72 h, they were used to create the combination treatment.

### 2.2. The Effect of the Combination of ET and ES

**Figure 4:**
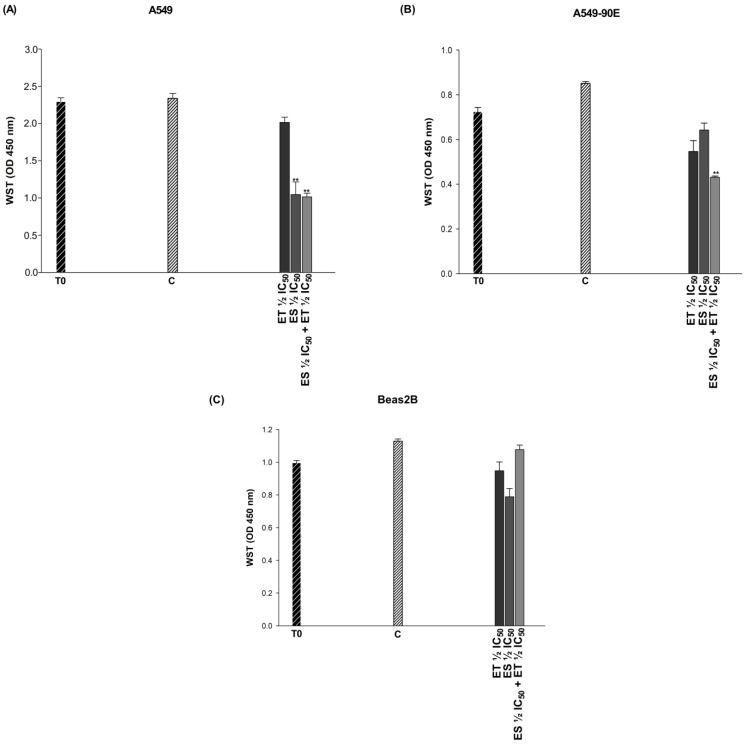
Cytotoxic effects of combinations exhibiting synergistic effects in A549 (**A**) and A549/90E (**B**) lung cancer cells and antagonistic effects in BEAS-2B (**C**) epithelial cells. The statistical analysis of the data was carried out by Student *t*-test. ** *p* < 0.01 were considered to indicate a statistically significant differences compared to control group.

The IC_50_ values of 48.67 and 51.6 μg/mL obtained for ET and ES, respectively, at the end of 24 h for A549 cells were applied reciprocally for each cell by including BEAS-2B together with the 2xIC_50_ and ½ IC_50_ (Table 2). The results of each combination were statistically evaluated with combination indices (CI) obtained using the Compusyn synergistic effect analysis program. Combination doses with a synergistic effect in A549 and A549/90E cells and an antagonistic effect in BEAS-2B cells have been determined (Table 3), and the cytotoxicity of the determined combinations has been plotted (Figure 4). The effects of all tested combinations are given in Table S2 and Figures S3–S5. The effects of the drugs alone and in combination on cell viability at doses determined to be antagonistic in BEAS-2B cells but synergistic in cancer cells were calculated using the following formula: 1-[(ODtreatment/OD control) × 100]. ET ½ IC_50_, ES ½ IC_50_, and the ET ½ IC_50_ + ES ½ IC_50_ combination caused 18.37%, 55.19%, and 57.55% death in A549 cells whereas they caused 44.9%, 22.4%, and 51.94% death in A549/90E cells, respectively.

### 2.3. Colorimetric Assays (Caspase3, P-gP, PTEN)

**Figure 5:**
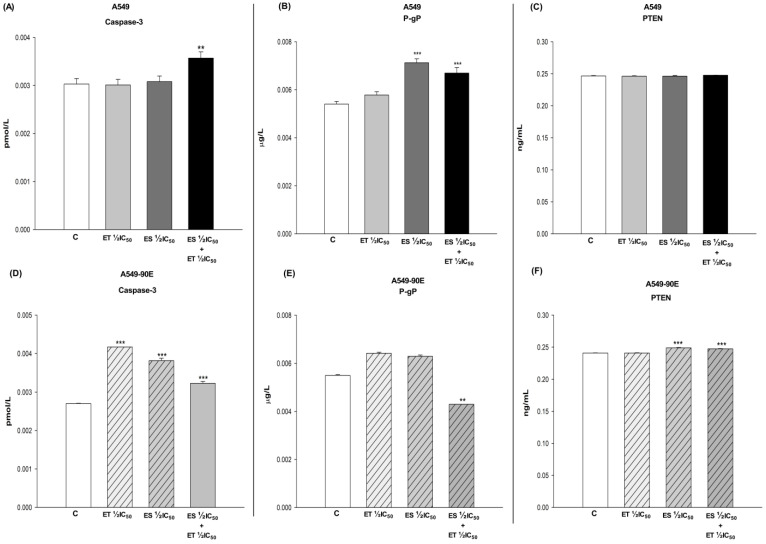
Amounts of caspase3 (**A**,**D**), P-gP (**B**,**E**), and PTEN (**C**,**F**) in A549 and A549/90E cells after ET ½ IC_50_, ES ½ IC_50_, and ET ½ IC_50_ + ES ½ IC_50_ treatment. The data obtained from the caspase activity assays were evaluated using the one-way ANOVA test and Mann–Whitney U test in the Graph-Pad InStat statistical program. ** *p* < 0.01 and *** *p* < 0.001 were considered to indicate a statistically significant differences compared to control group.

The doses determined to be synergistic in cancer cells and antagonistic in BEAS-2B cells were applied to A549 and A549/90E cells alone and in combination. The effects of ET ½ IC_50_, ES ½ IC_50_, and the ET ½ IC_50_ + ES ½ IC_50_ combination on the amount of caspase-3, P-gP, and PTEN were evaluated colorimetrically. Changes in caspase-3, P-gP, and PTEN levels induced by the drugs alone and in combination were determined by direct comparison with untreated control cells (Figure 5). In A549 cells, the combination of ES ½ IC_50_ and ET ½ IC_50_ caused increased levels of caspase-3 (*p* < 0.01) and P-gP (*p* < 0.001), while PTEN levels remained unchanged (*p* > 0.05). The combination resulted in an increase in caspase-3 (*p* < 0.001) and PTEN (*p* < 0.001) amounts alongside a decrease in P-gP (*p* < 0.01) levels in A549/90E cells.

### 2.4. Annexin V/PI Staining

**Figure 6:**
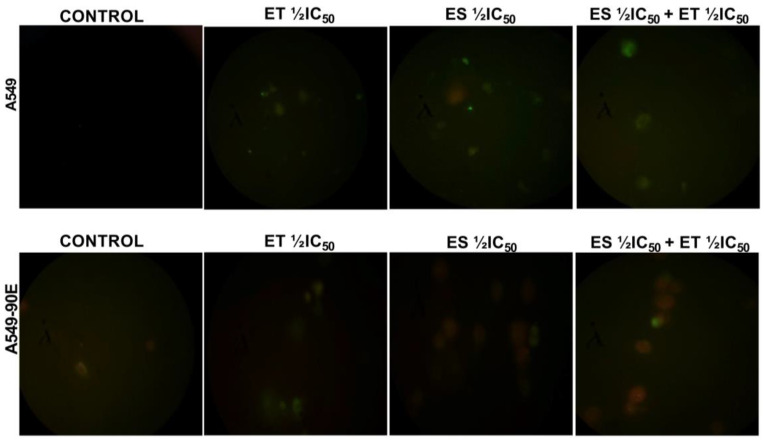
Analysis of apoptosis induced by ET ½ IC_50_, ES ½ IC_50_, and ET ½ IC_50_ + ES ½ IC_50_ treatment in A549 and A549/90E cells compared with untreated control cells after 24 h of incubation in a fluorescent microscope. This assay identifies apoptotic cells based on Annexin V binding, distinguishing between early apoptosis (Annexin V positive, PI negative) and late apoptosis (both Annexin V and PI positive) using the Elabscience E-CK-A211 kit (scale bar is 100 μm with ×40 magnification).

The translocation of phosphatidylserine (PS) from the inner to the outer leaflet of the cytoplasmic membrane is a hallmark of apoptosis, differentiating it from necrosis. The Annexin V-FITC kit was used to find out if the cell death caused by the drugs alone (ET ½ IC_50_ and ES ½ IC_50_) and in combination (ET ½ IC_50_ + ES ½ IC_50_) was apoptotic or not. In accordance with the kit procedure, the stained cells were analyzed using a fluorescence microscope, and the images were recorded. Cells giving green fluorescence were considered apoptotic, and cells giving red fluorescence due to PI were considered necrotic (Figure 6).

### 2.5. Mitochondrial Membrane Potential (ΔΨm)

**Figure 7:**
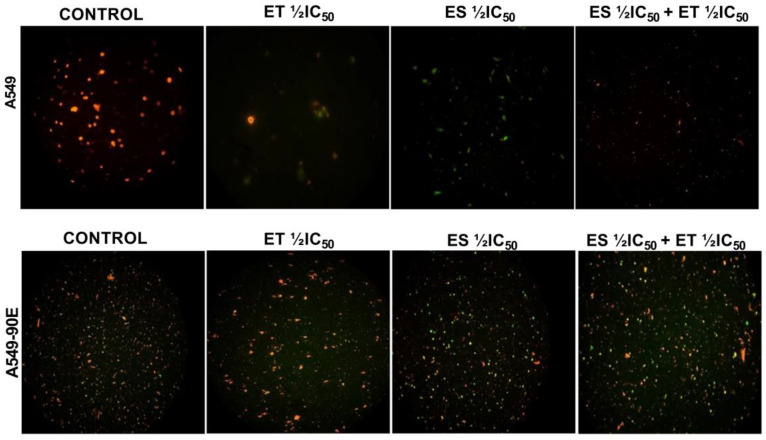
ET ½ IC_50_, ES ½ IC_50_, and ET ½ IC_50_ + ES ½ IC_50_ treatment-induced changes in mitochondrial membrane potential (ΔΨm) detected by JC-1 staining in A549 and A549/90E cells compared with untreated control cells after 24 h were visualized, and images were captured using a fluorescence microscope (scale bar is 100 μm with ×40 magnification).

Considering that mitochondrial membrane potential (ΔΨm) has a critical role in cell apoptosis, we determined the changes in ΔΨm in A549 and A549/90E cells after treatment with ET ½ IC_50_, ES ½ IC_50_, and the combination (ET ½ IC_50_ + ES ½ IC_50_). Competent mitochondria accumulate red fluorescent J-aggregates, whereas green fluorescence of the JC-1 dye signifies reduced mitochondrial membrane potential, indicating that cells are undergoing apoptosis. As shown in Figure 7, in both drugs, alone and in combination, treated cells’ green fluorescent intensity increased when compared with untreated control cells.

### 2.6. Target Gene Prediction of ES, ET, and Lung Cancer

A total of 58, 182, and 650 target genes were identified in the DgIdb and CTD databases for ES, ET, and lung cancer, respectively (Supplementary Tables S3–S5).

### 2.7. Screening of Potential Lung Cancer Related Target Genes of ES and ET

**Figure 8:**
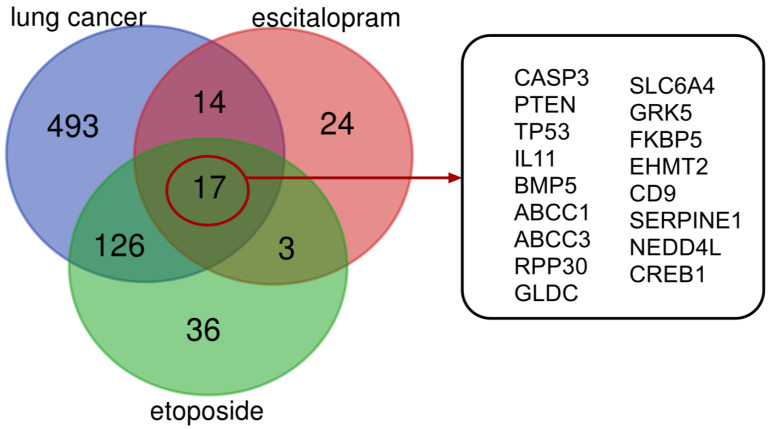
Association of target genes via Venn diagram between ET and ES against lung cancer. There are 31 target genes in ET + ES intersection against lung cancer. Biotargets that are shared by ET, ES, and lung cancer are 17 in total.

The Venn diagram (Figure 8) illustrates the possible lung cancer-related target genes of ES and ET. The diagram reveals 31 ES intersection, 143 ET intersection, and 157 ES + ET intersection target genes related to lung cancer. The online databases identified 17 shared biotargets of ES and ET against lung cancer.

### 2.8. PPI Network

**Figure 9:**
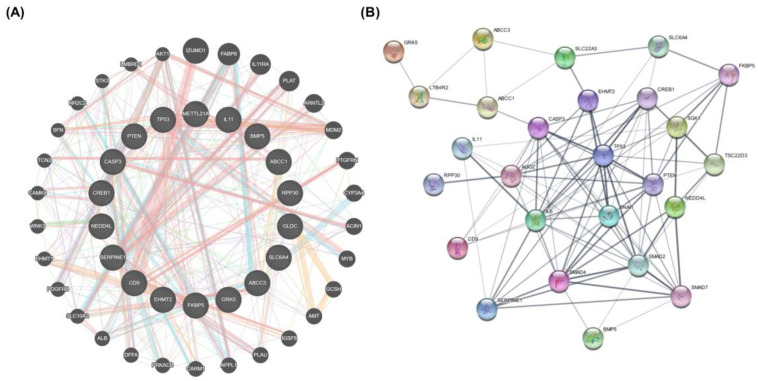
STRING protein interaction network and GeneMANIA gene interaction network. (**A**) GENEMANIA database results. Interaction network of the 17 genes in this network between 30 potential target genes in terms of physical and genetic interactions, co-localization, and co-expression levels. (**B**) Protein–protein interaction network (PPI) of intersection target genes of shared biotargets against lung cancer using the STRING database.

In this study, we examined the intersection of ES, ET, and lung cancer. The PPI network of ES and ET sharing biotarget intersections against lung cancer was generated using STRING and GENEMANIA database (Figure 9). The networks demonstrated that these target genes exhibit complex interactions among themselves. Several potential target genes of ES and ET associated with lung cancer were also identified in the network and were considered as hub genes based on their node degree values. TP53, SERPINE1, PTEN, CASP3, CREB1, CD9, and ABCC1 were identified as hub genes overlapping in the intersection network. Identified hub genes and node degrees are given in Table 4.

### 2.9. GO and Pathway Enrichment Analysis

**Figure 10:**
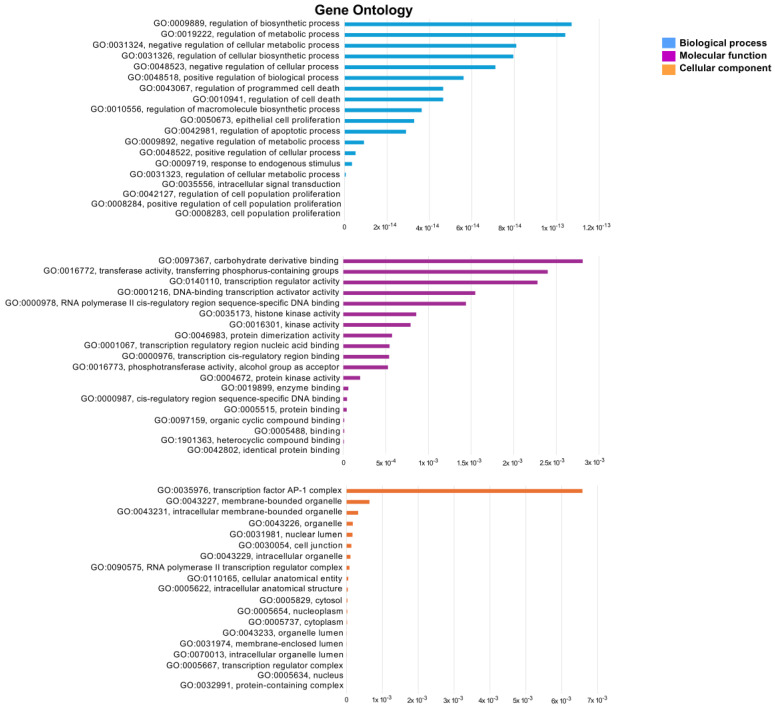
GO enrichment terms analysis. Top 20 significant enrichments represented by the biological process, cellular component, and molecular function of the intersection target genes of ET and ES against lung cancer.

GO enrichment analysis was conducted to further examine the biological processes, cellular components, and molecular functions related with the lung cancer target of ES and ET, utilizing DAVID and CTD bioinformatics resources. The GO analysis results for the top 20 biological processes inside the intersecting network indicated that the regulation of cellular biosynthetic processes and apoptosis is significantly more regulated than other processes. In the cellular component enrichment analysis, the top 20 significant enrichment terms included mainly the AP-1 complex and organelles among the intersection network. The twenty most significant enrichment terms pertaining to molecular function indicated that carbohydrate derivative binding and transferase activity were strongly regulated (Figure 10).

## 3. Discussion

Today, researchers are trying a wide range of methods to treat cancer. Off-label use of antidepressants widely reduces chronic pain in cancer and combats depression. Therefore, researchers decided to explore the efficacy of antidepressants in anticancer studies [63]. Patients diagnosed with cancer experience many intense emotional and behavioral problems, such as fear of death and fear of not being cured, with the uncertainties that arise during the treatment and palliation periods. Patients diagnosed with cancer exhibit a higher prevalence of depression than those with other medical disorders [64]. SSRIs, a class of new-generation antidepressants known for their improved tolerability and reduced adverse effects, are extensively utilized to address depression in patients receiving chemotherapy within oncology facilities [53].

ET, one of the first-line chemotherapy drugs used in lung cancer, triggers the formation of breaks in double-stranded DNA by inhibiting the enzyme topoisomerase II [65]. Unrepaired chain breaks initiate the DNA damage response and cause cell death. However, cells continue to proliferate in the presence of ET-induced stress by escaping apoptosis and developing drug resistance. The emergence of resistance to chemotherapeutic agents constitutes an important obstacle in the treatment of lung cancer. Up-regulation of ABCB1/MDR1/p-glycoprotein was detected in ET-resistant small cell lung cancer cells [66]. Even though chemotherapy is a common and effective way to treat cancer, further investigations are required to reduce the possible side effects of chemotherapeutics on normal cells, to prevent cellular resistance to chemotherapeutic agents, and to enhance the selective cytotoxic efficacy of current chemotherapeutics [67]. Numerous studies indicate that ET demonstrates significant efficacy in combination therapies and promotes apoptosis [21,65,68,69,70,71]. ET exhibited cytotoxic effects on both cancer and epithelial cells at all incubation times in the dose range we tested. Considering our cytotoxicity results, the damage caused by ET to a normal epithelium is quite clear. Although ET exhibited cytotoxic effects on cells at all three incubation times, the IC_50_ value was only detected in A549 cells after 24 h of incubation; no IC_50_ value was detected in the dose range tested in cells after 48 and 72 h of incubation. On the other hand, in A549/90E cells, which we rendered resistant, the IC_50_ value was not determined after 24 and 48 h of incubation; this value was determinable only after 72 h of incubation. This result proves that A549 cells become resistant to ET treatment. In BEAS-2B epithelial cells, similar to A549 cells, the IC_50_ value could be determined only after 24 h of incubation. After 24 h of incubation, the IC_50_ values obtained for ET in A549 and BEAS-2B cells were 48.67 and 79.38 μg/mL, respectively. The dose of 48.67 μg/mL found by ET’s cytotoxic activity on A549 cells was lower than that found in BEAS-2B cells. This meant that the dose of 48.67 μg/mL for ET had to be chosen to be selective.

ES has been shown to exhibit cytotoxic effects in different cancer cells [72,73]. Chen et al. reported that ES induced apoptosis by increasing the expression of apoptotic proteins Bax, cytochrome c, apaf-1, caspase-3, caspase-9, and PARP in U-87MG cells in vitro and in vivo [74]. Furthermore, ES has been shown to induce apoptosis in A549 and H460 non-small cell lung cancer cells through the activation of mitochondria-dependent apoptotic signaling pathways. ES elevated the protein expression levels of the apoptosis regulator Bax, tBid, cytochrome c, Apaf1, cleaved caspase-3, and caspase-9 [15]. Herein, we studied the cytotoxic effects of ES, alone and in combination with ET, on A549 cells and for the first time on A549/90E. ES exhibited cytotoxic effects on A549 cells throughout all three incubation periods; however, the IC_50_ value was determined to be 51.6 μg/mL in A549 and 99.71 μg/mL in A549/90E cells only following 24 h of incubation. The data acquired from our investigation align with the results derived from the cytotoxicity assays conducted by Yuan et al. in A549 cells [15]. The cytotoxic effect exhibited by ES in A549/90E cells suggests that ES may have modified the expression and/or function of certain proteins associated with resistance mechanisms in these cells. Furthermore, the IC_50_ value for ET in BEAS-2B cells after 24 h of incubation was found to be 79.38 μg/mL, while it was 165.42 μg/mL for ES. This result clearly shows that ES was not cytotoxic for BEAS-2B cells, neither in A549 cells nor in A549/90E cells at their IC_50_ values; in other words, it did not cause even 50% cell death.

Combinations of ES and chemotherapeutic agents have been studied in various cancer cell lines [75]. However, there are a limited number of studies evaluating the cytotoxic effects of ES combinations on lung cancer cells. The cytotoxic effects of the ES and ET combination in this study clearly demonstrate that the doses used in the combination are crucial when targeting a selective cytotoxic effect on cancer cells. Figure 3 demonstrates that the combinations do not induce cytotoxic effects on BEAS-2B cells when supplied at appropriate dosages. Consequently, the essential aspect of our research is that we concentrated on doses that did not induce cytotoxicity in BEAS-2B lung epithelial cells, while demonstrating considerable cytotoxic effects in A549 and A549/90E cells. Since the cytotoxic effects of the ES ½ IC_50_ + ET ½ IC_50_ combination were determined to be selective to both A549 and A549/90E cells, we further investigated the death mechanism caused by this combination by studying the changes in the amounts of caspase 3, PTEN, and P-gP proteins. Statistically significant increases in the amount of caspase-3 triggered by ES ½ IC_50_, ET ½ IC_50_, and the combination (ES ½ IC_50_ + ET ½ IC_50_) in both A549 and A549/90E cells suggest that the mechanism of death may be apoptosis. This suggestion was validated by Annexin V-FITC and ΔΨm assays. Specifically, the combination-induced cell death was found to be apoptosis, triggered by the intrinsic pathway. In addition, in A549/90E cells, ES ½ IC_50_ + ET ½ IC_50_ increased the amount of PTEN and decreased the amount of P-gP. The increase in PTEN, a tumor suppressor, and the decrease in P-gP suggest that combining ET with ES may help to treat cancer cells resistant to ET.

Several hub genes were identified based on the PPI network and considered to play a crucial role within the network against pancreatic cancer [76,77,78,79]. This study suggests that *TP53*, *SERPINE1*, *PTEN*, *CASP3*, CREB1, *CD9*, and *ABCC* may serve as key targets for the anticancer activity of the ES and ET combination, which may possess significant biological importance in the treatment of lung cancer. Mutated *TP53* facilitates the carcinogenesis of lung epithelial cells, significantly influencing the progression of lung cancer. It contributes to distant metastases, affecting the prognosis of lung cancer. Mutant *TP53* is recognized for exhibiting resistance to conventional chemotherapeutic agents, frequently rendering the disease untreatable [80]. *SERPINE1* has been identified as highly overexpressed in various tumor tissues. Elevated *SERPINE1* expression serves as a biomarker in various solid tumors, including breast cancer, ovarian cancer, colon cancer, and non-small cell lung cancer, and it is regarded as a possible therapeutic target [81]. *SERPINE1* has been shown to have pro-angiogenic, growth, migration stimulation, and anti-apoptotic activity, all of which are targeted to promote tumor growth, cancer cell survival, and metastasis [81,82]. Similarly, increased expression of *CREB1* has been associated with poor prognosis in lung cancer, and its suppression has been reported to be promising in treatment [83]. Our colorimetric data on PTEN, CASP3, and P-gP validate the significance of these genes identified through PPI network analysis. Consequently, these genes may serve as optimal targets for the combination of ES and ET in regulating lung cancer proliferation. Also in this study, the possible mechanism of action against lung cancer was further evaluated through enrichment analysis. The GO enrichment analysis revealed that the most enriched biological process among the target genes of the ES and ET combination pertained to the regulation of cellular biosynthesis and apoptosis. Therefore, it is important to delicately balance the regulation of the two signals to improve the efficacy of combination therapy between ES and ET. Targeting genes involved in the regulation of cell death could improve the treatment of lung cancer.

## 4. Materials and Methods

### 4.1. Cells and Culture Conditions

The A549 human lung cancer cell line (ATCC^®^ CCL-185™) and BEAS-2B lung epithelial cell line (ATCC ^®^ CRL-9609TM) from the American Type Culture Collection (ATCC) as well as A549 cells previously rendered resistant to etoposide in our laboratory (A549/90E) were used in this study. A549 and A549/90E cells were grown in Rosewell Park Memorial Institute 1640 medium (RPMI) with 10% fetal bovine serum (FBS) and 2% L-glutamine. BEAS-2B lung epithelial cells were grown in BEBM medium supplemented with 10% FBS. The cells were maintained at 37 °C in a humidified atmosphere of 5% CO2. Cells were passaged at a ratio of 1:2 or 1:3 by a mixture of 0.25% trypsin and 0.03% EDTA as recommended by ATCC. Cells that were not used during the experimental period were kept overnight at −20 °C in a solution containing 95% medium and 5% DMSO and then stored in a deep freezer at −80 °C and then in a liquid nitrogen tank (−196 °C).

### 4.2. Preparation of Etoposide Resistant A549 Cells

A broad spectrum of etoposide concentrations was tested to determine the appropriate starting dose that allowed growth of the etoposide-resistant A549 cell line. Graphs were created using the Sigma Plot 10.0 program with the data obtained after etoposide application, and IC_50_ values were calculated. Using IC_50_, the cytotoxic effects (cytotoxic index) that a drug will exhibit in any cell line can be determined. In this way, resistance to the drug can also be determined. According to the study by Hansen et al. (2003), the cellular response to the treatment was assessed, categorizing cells with a cytotoxic index > 50% (IC_50_) as drug-sensitive and those with <50% as drug-resistant [84]. To determine the initial cytotoxic dose of ET in the A549 cell line, cells were treated with diluted concentrations of ET starting from 1000 μg/mL up to 1.95 μg/mL. Three sets of experiments were prepared, each with four replicates. Each set of experiments was terminated with the CCK-8 solution at the end of 24, 48, and 72 h of incubation. As a result of the calculations, when two Petri dishes of A549 cells expanded from the stock reached at least 80% confluence, 5 μg/mL ET prepared in 10% serum containing medium was applied to both Petri dishes. After each 24 h incubation period, the cells were examined under a microscope and the medium was refreshed with ET-medicated 10% medium. As a result of frequent microscope observations, it was observed that the occupancy of the Petri dishes decreased rapidly to approximately 20%. Cells that stabilized at this occupancy for a certain period of time were considered to have become resistant to the ET-medicated medium and were left to incubate to increase their numbers. When the occupancy of the Petri dishes reached approximately 80%, the old medium was replaced by doubling the concentration of ET to 5 μg/mL. As a result of the observations, the Petri dishes, which were rapidly becoming less full, were again refreshed with the medium containing a higher concentration of the drug after 24 h of incubation. After a while, the cells developed resistance to this drug concentration and started to increase their numbers. After approximately 6 months of these experiments, when both Petri dishes were 80–90% full, it was decided that resistance was successfully established. This new A549 cell line, which became resistant to 90 μg/mL ET, was named A549/90E. Cells were treated with a mixture of 0.25% trypsin and 0.03% EDTA and passaged at a 1:2 ratio. Following passaging, the new generation of A549/90E cells were adequately stocked for further use in the experiments.

### 4.3. Cell Proliferation (CCK-8) Assay

The cells were seeded at 5 × 10^3^ cells per well in 100 µL complete medium onto 96-well plates. After 24 h of incubation, the medium was removed. At the end of the incubation period, dose screening for different concentrations of ES (5, 10, 25, 50, 100, 200, 250, 400, and 500 μg/mL) and ET (5, 25, 50, 100, 250, and 500 ng/mL) prepared in medium containing 1% serum was performed separately in 96-well sterile plates in four replicates. Immediately after drug administration, a time-zero reading was taken to determine the initial cell number. Following drug treatments, cells were incubated for 24, 48, and 72 h. At the end of each incubation period, the experiment was terminated, and 10 μL of CCK-8 (Abbkine, Atlanta, CA, USA) solution was added and left to incubate for more 3 h. At the end of the incubation period, the absorbance values of the plates were measured and recorded on an Elisa reader (Thermo Scientific Multiskan Go, Thermolabsystem, Chantilly, VA, USA) at 450 nm wavelength [85]. The results of three independent experiments, each with 8 replicates, were statistically evaluated and compared with the control group, and the IC_50_ values of each drug were calculated for all incubation times.

### 4.4. Trypan Blue

For direct microscopic determination of cell viability, the trypan blue (0.4%; in Hanks salt-phosphate buffer) assay was used with minor revision. Cells were seeded onto 24-well plates at 5 × 10^4^ cells/well. After 24 h of incubation, the media were removed; 1% serum medium containing the drugs prepared at the IC_50_ values determined after dose screening and only 1% serum medium were applied to the wells used as control group. After the incubation periods were completed, the media were collected and precipitated by centrifugation at 1000 rpm for 2 min. Next, 200 μL of EDTA-Versen was added to the wells, and the cells were collected from the plates and added to the precipitate in the tube. The mixture was centrifuged at 2500 rpm for another 5 min to precipitate. The supernatant was removed, and the pellets were thawed by adding 250 μL trypan blue and 250 μL medium containing 1% serum. The samples were placed on ice and diluted depending on the cell concentration, and the number of live and dead cells were determined under a light microscope using a hemocytometer [86].

### 4.5. Neutral Red Assay

Cell viability was also assessed by neutral red uptake assay (Sigma-Aldrich, SaintQuentin-Fallavier, France) with minor revisions. Dry neutral red (NR) (1 mg) was dissolved in 10 mL PBS, and 100 mg/mL (2500×) stock solution was obtained. An intermediate stock solution of 4 mg/mL (100×) was prepared by dissolving 0.5 mL of the stock solution in 12 mL PBS. Neutral red working solution was prepared one day in advance for each use and incubated overnight in the incubator. Afterwards, 0.5 mL of 4 mg/mL intermediate stock NR solution was combined with 49.5 mL of medium containing 1% serum to prepare a sufficient amount of working solution. Each well was washed by adding 100 μL PBS. Then, 150 μL of NR working solution was added to each well and incubated for 3.5 h. Finally, 100 μL of acidified ethanol solution (50% ethanol, 1% acetic acid, 49% H_2_O) was added and incubated for 15 min in the dark at room temperature. Absorbance values of the plates were measured at 540 nm using a microplate reader (Thermo Scientific Multiskan Go, Thermolabsystem, Chantilly, VA, USA) [87].

### 4.6. CompuSyn Combination Analysis

The obtained data were evaluated in CompuSyn (Version 1.0), a software program based on the “efficiency calculation of combination in multiple drug interactions” prepared by Chou and Talalay [88]. The CompuSyn program, as the name suggests, is prepared to evaluate the possible synergism of two drugs. Upon calculation, if the combination index (CI) value equaled 1, the drugs were deemed additively effective; if it was greater than 1, they were considered antagonistically effective; and if it was less than 1, they were regarded as synergistically effective.

### 4.7. Quantification of Caspase-3

Caspase-3 amounts were determined following the protocol of the caspase-3 colorimetric ELISA kit (Abbkine, KTE62614, CA, USA). Briefly, cells were seeded onto 100 mm Petri dishes at 5 × 10^4^ cells as recommended in the kit protocol. After dose screening, the drugs prepared in medium containing 1% serum at the determined IC_50_ values and effective combinations were applied 8 mL per Petri dish, each with three replicates. At the end of the incubation period, the medium was removed, and the cells were collected using a cell scraper. The collected cells were centrifuged at 1000 g for 25 min. After five freeze–thaw steps, the cells were centrifuged at another 1000 g for 20 min, and the supernatants were transferred. The protein concentration of the samples was determined with the commercial Pierce ^TM^ Protein Assay Kit (Thermo Scientific, No:23227, Waltham, MA, USA) and measured against bovine serum albumin standards. Samples of the cell lysates were seeded into a standard 96 well-plate, as each sample contains 10 µg protein per well, and the amount of caspase-3 in the supernatants was determined following the kit protocol. Absorbance values were measured and recorded on a microplate reader (Thermo Scientific Multiskan Go, Thermolabsystem, Chantilly, VA, USA) at 450 nm wavelength [89].

### 4.8. Quantification of PTEN

PTEN amounts were determined following the protocol of the PTEN colorimetric ELISA kit (BTLAB, E3242Hu, Birmingham, UK). Cells were seeded onto 100 mm Petri dishes at 5 × 10^4^ cells as recommended in the kit protocol. After dose screening, the drugs prepared in medium containing 1% serum at the determined IC_50_ values and effective combinations were applied 8 mL per Petri dish, each in triplicate. At the end of the incubation period, the medium was removed and the cells were collected using a cell scraper. The collected cells were centrifuged at 1000 g for 25 min. After five freeze–thaw steps performed, the cells were centrifuged at another 1000 g for 20 min and the supernatants were transferred to new Eppendorfs. Protein amounts were equalized, and the amount of PTEN in the supernatants was determined following the kit protocol. Absorbance values were measured and recorded on a microplate reader (Thermo Scientific Multiskan Go, Thermolabsystem, Chantilly, VA, USA) at 450 nm wavelength.

### 4.9. Annexin V Fluorescein Isothiocyanate (FITC)/PI Apoptosis Assay

Annexin V and propidium iodide (PI) staining are used to detect apoptotic cell death. Annexin V shows a strong binding affinity for phosphatidylserine in the presence of Ca^2+^. PI has the ability to bind to DNA and stains only necrotic or late apoptotic cells [90]. Apoptotic cells were analyzed according to the protocol of the the Annexin V FITC/PI Apoptosis detection kit (Elabscience, E-CK-A211). Apoptosis of cells, treated with the respective agents, was induced. After completion of the incubation, the cells were detached from the plates with a cell scraper. The collected cells were centrifuged at 300 g for 5 min, and the supernatant was discarded. PBS was added to wash the cells and cells were gently resuspended. The cell suspension was divided into tubes of 2.5 × 10^5^ cells each and centrifuged at 300 g for 5 min, and the supernatant was discarded. PBS was added to wash the cells and discard the supernatant. Later, the cells were suspended in 100 µL binding buffer mixed with 5 µL Annexin V FITC and 5 µL PI stain. Then, the cells were gently vortexed and incubated for 15 min at room temperature in the dark. Finally, 400 µL binding buffer was added to each sample and placed on ice in the dark. The stained cells were imaged using a fluorescent microscope (Nikon Eclipse E200, Tokyo, Japan) within 1 h [91,92].

### 4.10. Mitochondrial Membrane Potential (ΔΨm)

Cationic carbocyanine dye-1 (JC-1) is used to distinguish healthy and apoptotic cells by indirectly measuring the mitochondrial membrane potential (Abbkine, KTA4001). Cells were seeded into 6-well plates at 2.5 × 10^4^ cells/mL. After 24 h, the medium was removed, and the drugs prepared in 1 mL of medium containing 1% serum were applied. Three different control groups were used in the experiment: control negative, control positive, and no JC-1 stain. After the incubation of the drugs was completed, the kit protocol was followed: 2 mLof 1% medium was added to each of the 6-well plates treated with the current drug and cells were removed with a scraper. A total volume of 3 mL was collected into 15 mL falcons. The collected cells were centrifuged at 300 g for 5 min, and the supernatant was discarded. The pellets were dissolved with 1 mL of medium containing 1% serum. To the flask labeled as control positive, 1 μL CCCP was added and incubated at 37 °C for 10 min. Next, 10 μL of JC-1 dye was added to all falcons except the falcon labeled as unstained control and incubated at 37 °C for 40 min. At the end of the time, the cells were centrifuged at 300 g for 5 min at +4 °C. The supernatant was discarded, and 500 μL of the phosphate buffer in the kit was added, and the pellet dissolved. The samples were placed on ice and visualized using a fluorescent microscope (Nikon Eclipse E200, Japan) within 30 min [93,94].

### 4.11. Human P Glycoprotein Test

The amount of P-gP in the cells was determined following the protocol of the P-gP colorimetric ELISA kit (Abbkine, KTE60851). For P-gP quantification, cells were seeded onto 100 mm Petri dishes at 1 × 10^6^ cells/mL as recommended in the kit protocol. After dose screening, the drugs prepared in medium containing 1% serum at the determined IC_50_ values and effective combinations were applied 8 mL per Petri dish, each with three replicates. At the end of the incubation period, the medium was removed and the cells were collected using a cell scraper. The collected cells were centrifuged at 1000 g for 25 min. After five freeze–thaw steps, the cells were centrifuged at another 1000 g for 20 min, and the supernatants were transferred. Protein amounts were equalized, and the amount of P-gP in the supernatants was determined following the kit protocol. Absorbance values were measured and recorded on a microplate reader (Thermo Scientific Multiskan Go, Thermolabsystem, Chantilly, VA, USA) at 450 nm wavelength.

### 4.12. Statistical Analysis

The difference between the control and other groups in the experimental results obtained from cytotoxicity tests was evaluated using the Graph-Pad Prism version 4.00 (Graph-Pad Software, San Diego, CA, USA) statistical program, as well as the one-way ANOVA test followed by Dunnett’s multiple comparison test. The data obtained from the caspase activity assays were evaluated using the one-way ANOVA test and Mann–Whitney U test in the Graph-Pad InStat statistical program. IC_50_ values of the drugs were determined using the Sigma Plot 10.0 statistical program. All data were plotted as mean ± SEM values using the Sigma Plot 10.0 program.

### 4.13. Prediction of Drug–Target Gene Interactions

The target genes of ES and ET were retrieved from the two databases, which are the Drug Gene Interaction Database, DgIdb (https://www.dgidb.org/, (accessed on 17 January 2025)) [95], and the Comparative Toxicogenomics Database, CTD (http://ctdbase.org/, (accessed on 17 January 2025)) [96]. All target genes were restricted to *Homo sapiens* L., and duplicate target genes were excluded from the list.

### 4.14. Prediction of Lung Cancer Target Genes

Lung cancer-associated target genes were derived from the Online Mendelian Inheritance in Man, OMIM (https://www.omim.org/, (accessed on 17 January 2025)) [97], database, and “lung cancer” was used as the keyword.

### 4.15. Construction of Venn Diagram

All the predicted target genes for ES, ET, and lung cancer were imported into Bioinformatics and Evolutionary Genomics (http://bioinformatics.psb.ugent.be/webtools/Venn/, (accessed on 17 January 2025)) to identify the lung cancer-related target genes of ES and ET. The Venn diagram generated by the tool illustrates the intersection of potential target genes between the drug and the disease [45].

### 4.16. PPI Network Analysis

The protein–protein interaction (PPI) network was constructed using the Search Tool for Retrieval of Interacting Genes, STRING version 11.0 (https://string-db.org/, accessed on 17 January 2025), and GeneMANIA databases [98], inputting the target genes of ES and ET in relation to lung cancer to enhance the comprehension of protein interactions. The PPIs were imported into Cytoscape software, version 3.8.2 (https://cytoscape.org/ (accessed on 17 January 2025)) [99], an open-source tool for visualizing and integrating complex interaction networks alongside various attribute data [100] for the identification of hub genes. Each node in the network was evaluated and graded based on its “degree” value, with nodes exhibiting above-average degree values classified as hub compounds.

### 4.17. GO and Pathway Enrichment Analysis

The Database for Annotation, Visualization, and Integrated Discovery, DAVID, version 6.8 (https://david.ncifcrf.gov/home.jsp, (Accessed 25 February 2025)) [101], online software that offers comprehensive data for high-throughput gene functional analysis in the context of elucidating biological properties, was used to perform Gene Ontology (GO) enrichment analysis. The enrichment analysis was performed to elucidate the underlying mechanisms of the combination of ES and ET in combating lung cancer, focusing on biological processes, cellular components, molecular activities, and critical signaling pathways.

## Figures and Tables

**Table 1:** IC_50_ values obtained for ET and ES.

Drug	Cell	Time	IC_50_ (μg/mL)	Drug	Cell	Time	IC_50_ (μg/mL)
ET	A549	24 h	48.67	ES	A549	24 h	51.6
	A549-90E	72 h	499.82		A549-90E	72 h	99.71
	Beas2B	24 h	79.38		Beas2B	24 h	165.42

**Table 2:** Demonstration of reciprocal application of ET and ES combinations.

	ET			
ES		2xIC_50_	IC_50_	½ IC_50_
	2xIC_50_	ES 2xIC_50_ +ET 2xIC_50_	ES 2xIC_50_ + ET IC_50_	ES 2xIC_50_ + ET ½ IC_50_
	IC_50_	ES IC_50_ +ET 2xIC_50_	ES IC_50_ + ET IC_50_	ES IC_50_ +ET ½ IC_50_
	½ IC_50_	ES ½ IC_50_ +ET 2xIC_50_	ES ½ IC_50_ +ET IC_50_	ES ½ IC_50_ +ET ½ IC_50_

**Table 3:** CompuSyn Analysis of ET and ES combinations in A549, A549/90E, and BEAS-2B cells.

Cell	Dose	CI	Descrıptıon
A549	ES ½ IC_50_ + ET ½ IC_50_	0.66569	Synergism
Beas-2B	ES ½ IC_50_ + ET ½ IC_50_	2.5265	Antagonism
A549/90E	ES ½ IC_50_ + ET ½ IC_50_	0.34765	Synergism

**Table 4:** Hub genes affected by ET and ES in lung cancer.

Gene	İdentifier	Node Degree
ABCC1	9606.ENSP00000382342	3
CASP3	9606.ENSP00000311032	6
CD9	9606.ENSP00000371958	3
CREB1	9606.ENSP00000387699	5
PTEN	9606.ENSP00000361021	6
SERPINE1	9606.ENSP00000223095	5
TP53	9606.ENSP00000269305	7

## Data Availability

The data presented in this study are available in article.

## Supplementary Materials

The following supporting information can be downloaded at: https://www.mdpi.com/article/10.3390/ph18040531/s1, Table S1. Evaluation of IC_50_ values of A549, A549/90E and BEAS-2B cells with trypan blue. Figure S1. Cytotoxic effect of etoposide on cells after 24, 48 and 72 h of incubation (A) A549 (B) A549/90E (C) BEAS-2B. Figure S2. Cytotoxic effect of escitalopram on cells after 24, 48 and 72 h of incubation (A) A549 (B) A549/90E (C) BEAS-2B. Table S2. Efficacy Analysis of Escitalopram and Etoposide Combinations in A549, A549/90E and BEAS-2B Cells. Figure S3. Effects of ES and ET combinations on cell viability in A549 lines. Cell viability was assessed by CCK-8 and results were presented as optical density (OD450) values at 450 nm (** *p* < 0.01). Figure S4. Effects of ES and ET combinations on cell viability in A549/90E lines. Cell viability was assessed by CCK-8 assays and results were presented as optical density (OD450) values at 450 nm (** *p* < 0.01). Figure S5. Effects of ES and ET combinations on cell viability in BEAS-2B lines. Cell viability was assessed by CCK-8 and results were presented as optical density (OD450) values at 450 nm (** *p* < 0.01). Table S3. Target genes prediction of Escitalopram. Table S4. Target genes prediction of Etoposide. Table S5. Target genes prediction of lung cancer.
