# Supplementary material for: Cytotoxic Effect of Escitalopram/Etoposide Combination on Etoposide-Resistant Lung Cancer
Source: Pharmaceuticals (Basel). 2025 Apr 5;18(4):531. doi: 10.3390/ph18040531 (PMC12030030; doi:10.3390/ph18040531)
Supplement: Supplementary file 1 [file pharmaceuticals-18-00531-s001.zip › pharmaceuticals-3540210-supplementary.pdf]

Supplementary File

Cytotoxic Effect of Escitalopram/Etoposide Combination on Etoposide-Resistant Lung Cancer

Serap Özkaya Gül, Beyzanur Şimşek, Fidan Yıldız and Esra Aydemir *

Department of Biology, Faculty of Science, Akdeniz University, Antalya TR-07058, Turkey
* Correspondence: esra@akdeniz.edu.tr

1. Trypan blue assay

Table S1. IC_{50} values of ET and ES on A549, A549/90E and BEAS-2B cells with trypan blue

A549			A549/90E			BEAS-2B		
**Doses**	**24 hour**		**Doses**	**72 hour**		**Doses**	**24 hour**	
	**Mean**	**SEM +/-**		**Mean**	**SEM +/-**		**Mean**	**SEM +/-**
**Control**	97,600	0.6900	**Control**	98,919	0.1700	**Control**	98,045	0.3774
**ET: 49.2**	**(**)** 57.009	0.9697	**ET: 479.2**	**(**)** 53.102	0.6451	**ET: 61.65**	**(**)** 52.315	1,834
**ES: 55.8**	**(**)** 55.709	0.8522	**ES: 96.21**	**(**)** 55.731	0.7858	**ES: 200.52**	**(**)** 42.338	1,192

2. Neutral red assay

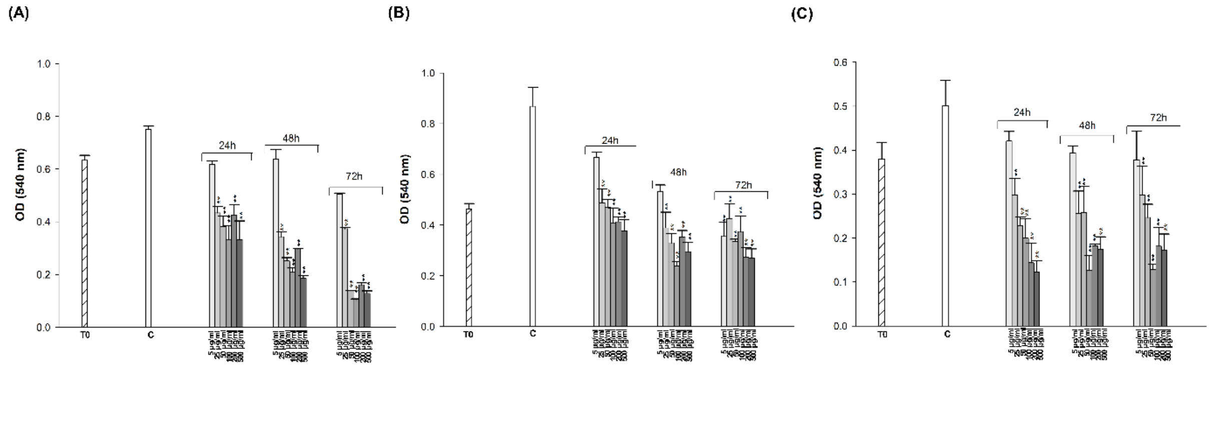

Figure S1. Cytotoxic effect of ET on cells after 24, 48 and 72 hours of incubation **(A)** A549 **(B)** A549/90E**(C)** BEAS-2B

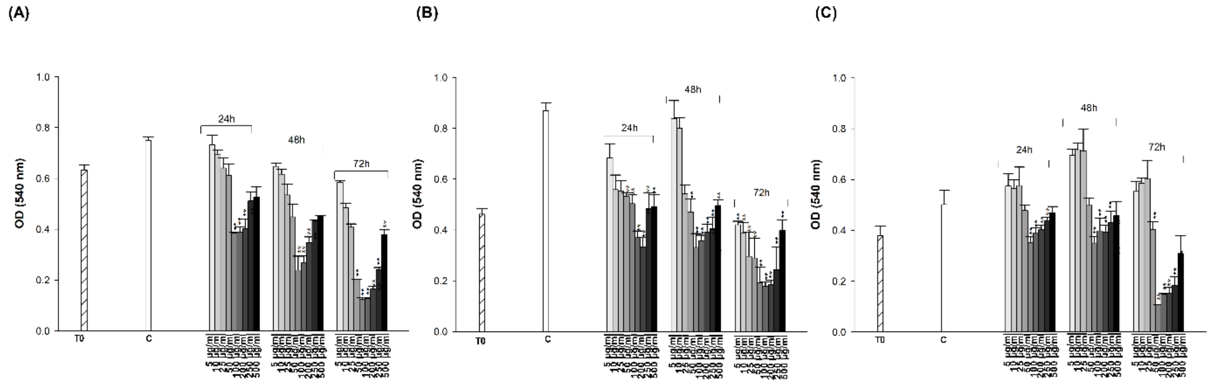

Figure S2. Cytotoxic effect of ES on cells after 24, 48 and 72 hours of incubation **(A)** A549 **(B)** A549/90E **(C)** BEAS-2B

Table S2. Efficacy Analysis of Escitalopram and Etoposide Combinations in A549, A549-90e and Beas2B Cells

Cell	Dose	CI	DESCRIPTION
**A549**	ES 2x IC_{50} + ET 2x IC_{50}	2.10027	Antagonism
	ES IC_{50} + ET 2x IC_{50}	5.70423	Strong Antagonism
	ES ½ IC_{50} + ET 2x IC_{50}	12.3525	Very Strong Antagonism
	ES 2x IC_{50} + ET IC_{50}	1.12015	Slight Antagonism
	ES IC_{50} + ET IC_{50}	1.64123	Antagonism
	ES ½ IC_{50} + ET IC_{50}	7.44727	Strong Antagonism
	ES 2x IC_{50} + ET ½ IC_{50}	4.58055	Strong Antagonism
	ES IC_{50} + ET ½ IC_{50}	1.23314	Moderate Antagonism
**Beas2B**	ES 2x IC_{50} + ET 2x IC_{50}	0.92003	Nearly additive
	ES IC_{50} + ET 2x IC_{50}	0.82969	Moderate Synergism
	ES ½ IC_{50} + ET 2x IC_{50}	1.78242	Antagonism
	ES 2x IC_{50} + ET IC_{50}	0.65461	Synergism
	ES IC_{50} + ET IC_{50}	0.62526	Synergism
	ES ½ IC_{50} + ET IC_{50}	1.3391	Moderate Antagonism
	ES 2x IC_{50} + ET ½ IC_{50}	0.63291	Synergism
	ES IC_{50} + ET ½ IC_{50}	0.55907	Synergism
**A549-90E**	ES 2x IC_{50} + ET 2x IC_{50}	2.2524	Antagonism
	ES IC_{50} + ET 2x IC_{50}	1.13939	Slight Antagonism
	ES ½ IC_{50} + ET 2x IC_{50}	2.53886	Antagonism
	ES 2x IC_{50} + ET IC_{50}	1.78937	Antagonism
	ES IC_{50} + ET IC_{50}	2.62958	Antagonism
	ES ½ IC_{50} + ET IC_{50}	2.12762	Antagonism
	ES 2x IC_{50} + ET ½ IC_{50}	1.67517	Antagonism
	ES IC_{50} + ET ½ IC_{50}	1.20789	Moderate Antagonism

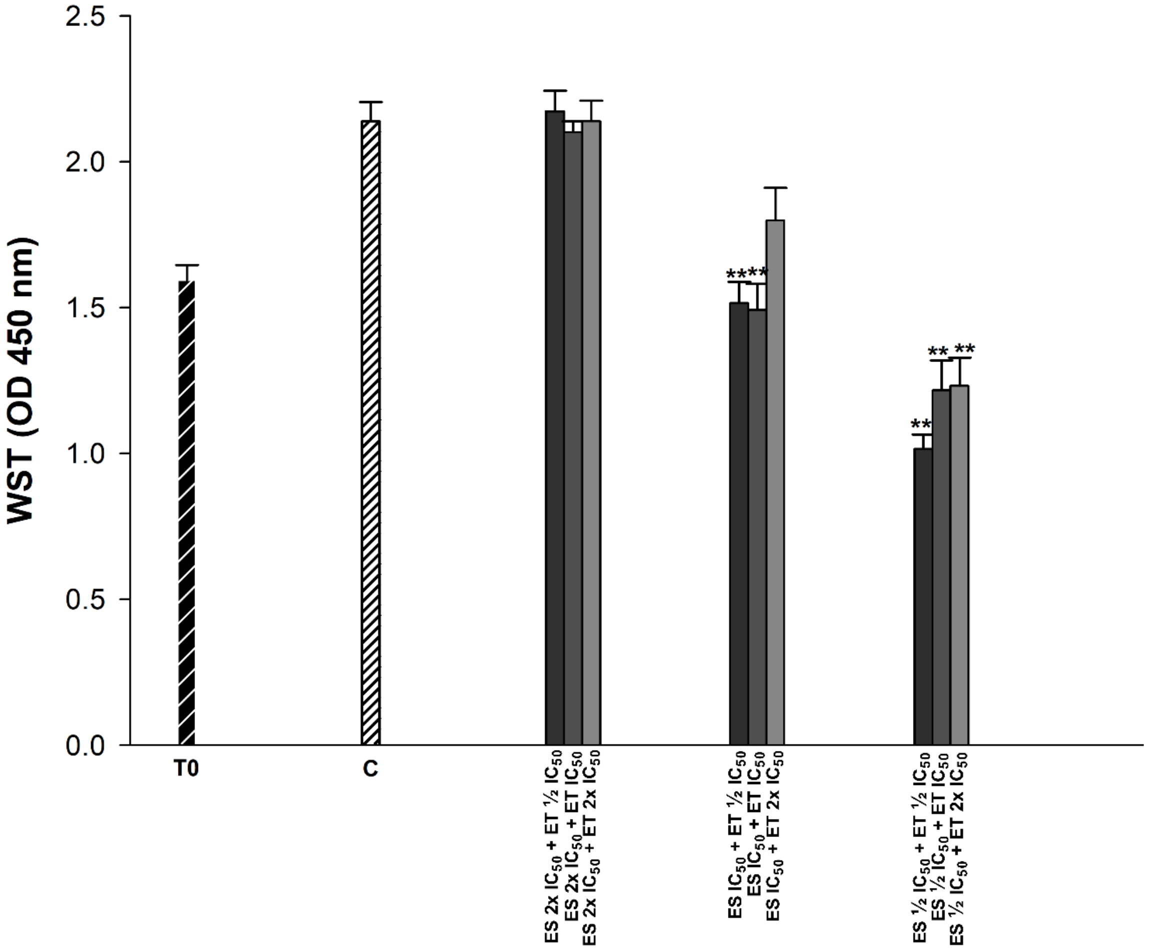

Figure S3. Effects of ES and ET combinations on viability of A549 cells. Cell viability was assessed by CCK-8 and results were presented as optical density (OD450) values at 450 nm (**P < 0.01).

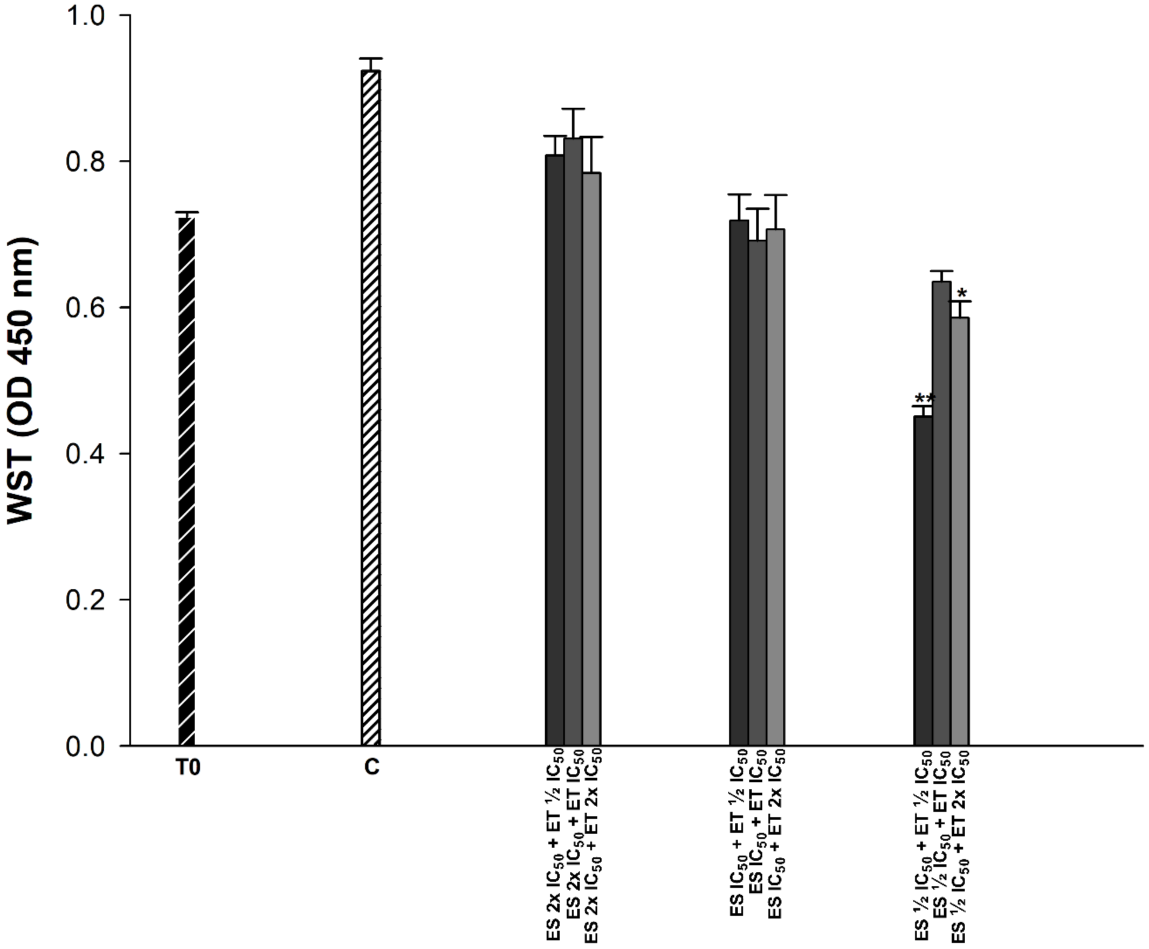

Figure S4. Effects of ES and ET combinations on viability of A549/90E cells. Cell viability was assessed by CCK-8 assays and results were presented as optical density (OD450) values at 450 nm (**P <0.01).

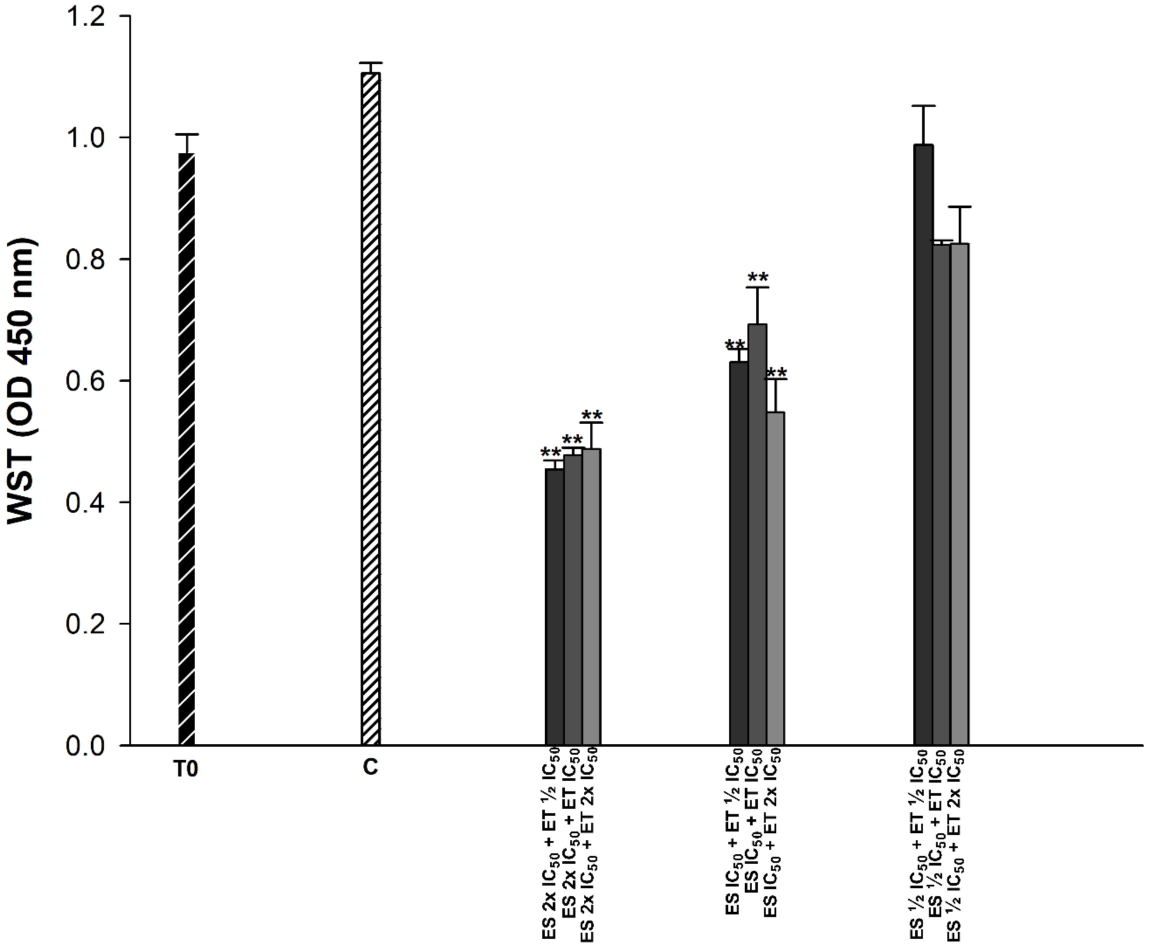

Figure S5. Effects of ES and ET combinations on viability of BEAS-2B cells. Cell viability was assessed by CCK-8 and results were presented as optical density (OD450) values at 450 nm (**$P < 0.01$).

Table S3. Target genes prediction of ES

Gene name	Drug Name	Data Sources 1	Data Sources 2
**FKBP5**	Escitalopram	DGIdb	CTD
**GLDC**	Escitalopram	DGIdb	CTD
**SLC6A4**	Escitalopram	DGIdb	CTD
**METTL21A**	Escitalopram	DGIdb	CTD
**CYP2C19**	Escitalopram	DGIdb	CTD
**DTNBP1**	Escitalopram	DGIdb	CTD
**ERICH3**	Escitalopram	DGIdb	CTD
**NEDD4L**	Escitalopram	DGIdb	CTD
**GRIA3**	Escitalopram	DGIdb	CTD
**BMP5**	Escitalopram	DGIdb	CTD
**PAPLN**	Escitalopram	DGIdb	CTD
**HTR1B**	Escitalopram	DGIdb	CTD
**COL26A1**	Escitalopram	DGIdb	CTD

**RORA**	Escitalopram	DGIdb	CTD
**GRIK4**	Escitalopram	DGIdb	CTD
**GSK3B**	Escitalopram	DGIdb	CTD
**GRIK2**	Escitalopram	DGIdb	CTD
**REEP5**	Escitalopram	DGIdb	CTD
**KDM4A**	Escitalopram	DGIdb	CTD
**CRHR2**	Escitalopram	DGIdb	CTD
**CYP2D6**	Escitalopram	DGIdb	CTD
**CD8B**	Escitalopram	DGIdb	CTD
**CACNA1C**	Escitalopram	DGIdb	CTD
**RFK**	Escitalopram	DGIdb	CTD
**CYP2C18**	Escitalopram	DGIdb	CTD
**IL11**	Escitalopram	DGIdb	CTD
**CD9**	Escitalopram	DGIdb	CTD
**EHMT2**	Escitalopram	DGIdb	CTD
**RPP30**	Escitalopram	DGIdb	CTD
**TPH1**	Escitalopram	DGIdb	CTD
**SERPINE1**	Escitalopram	DGIdb	CTD
**GRK5**	Escitalopram	DGIdb	CTD
**SRP19**	Escitalopram	DGIdb	CTD
**CREB1**	Escitalopram	DGIdb	CTD
**CYP2C9**	Escitalopram		CTD
**ABCC1**	Escitalopram		CTD
**PGP**	Escitalopram		CTD
**ABCC3**	Escitalopram		CTD
**TP53**	Escitalopram		CTD
**BCL2L1**	Escitalopram		CTD
**BIRC5**	Escitalopram		CTD
**CCND1**	Escitalopram		CTD
**CDKN1B**	Escitalopram		CTD
**DAPK1**	Escitalopram		CTD
**ERBB2**	Escitalopram		CTD
**FGF9**	Escitalopram		CTD
**FGFR1**	Escitalopram		CTD
**FOS**	Escitalopram		CTD
**HRAS**	Escitalopram		CTD
**JUN**	Escitalopram		CTD
**MYC**	Escitalopram		CTD
**PIK3CA**	Escitalopram		CTD
**RAF1**	Escitalopram		CTD
**STAT3**	Escitalopram		CTD
**E2F1**	Escitalopram		CTD
**E2F8**	Escitalopram		CTD
**FGF9**	Escitalopram		CTD
**FGFR1**	Escitalopram		CTD
**MDM2**	Escitalopram		CTD

**VEGFA**	Escitalopram		CTD

Table S4. Target genes prediction of Etoposide

Gene name	Drug Name	Data Sources 1	Data Sources 2
**TBP**	Etoposide	DGIdb	CTD
**AFP**	Etoposide	DGIdb	
**GSTM1**	Etoposide	DGIdb	
**HSPB2**	Etoposide	DGIdb	
**UGT1A10**	Etoposide	DGIdb	
**TOP2B**	Etoposide	DGIdb	
**GLP1R**	Etoposide	DGIdb	
**ALK**	Etoposide	DGIdb	
**ABCC1**	Etoposide	DGIdb	
**EIF4E**	Etoposide	DGIdb	
**SF3B1**	Etoposide	DGIdb	
**CYP1A2**	Etoposide	DGIdb	
**DDIT3**	Etoposide	DGIdb	
**BCL2**	Etoposide	DGIdb	
**BTF3P11**	Etoposide	DGIdb	
**CYP2D6**	Etoposide	DGIdb	
**PGP**	Etoposide	DGIdb	
**ATM**	Etoposide	DGIdb	
**TOP2A**	Etoposide	DGIdb	
**E2F1**	Etoposide	DGIdb	
**NCOA3**	Etoposide	DGIdb	
**BLMH**	Etoposide	DGIdb	
**CYP2C19**	Etoposide	DGIdb	
**YAP1**	Etoposide	DGIdb	
**MAPK3**	Etoposide	DGIdb	
**MYCN**	Etoposide	DGIdb	
**IGF2**	Etoposide	DGIdb	
**KLK3**	Etoposide	DGIdb	
**BAX**	Etoposide	DGIdb	
**NQO1**	Etoposide	DGIdb	
**TYMS**	Etoposide	DGIdb	
**SLIT1**	Etoposide	DGIdb	
**NR4A1**	Etoposide	DGIdb	
**SLCO1B1**	Etoposide	DGIdb	
**SLC2A4**	Etoposide	DGIdb	
**XIAP**	Etoposide	DGIdb	
**DYNC2H1**	Etoposide	DGIdb	
**ACTR2**	Etoposide	DGIdb	
**ITGAL**	Etoposide	DGIdb	
**ABCC3**	Etoposide	DGIdb	
**PLA2G1B**	Etoposide	DGIdb	
**MUC16**	Etoposide	DGIdb	
**CHEK1**	Etoposide	DGIdb	

**PLA2G6**	Etoposide	DGIdb	
**TGFB1**	Etoposide	DGIdb	
**MAPK1**	Etoposide	DGIdb	
**CYP2C9**	Etoposide	DGIdb	
**PAPOLA**	Etoposide	DGIdb	
**TYMSOS**	Etoposide	DGIdb	
**NCOA1**	Etoposide	DGIdb	
**GBX2**	Etoposide	DGIdb	
**TNFSF13B**	Etoposide	DGIdb	
**FGFR1**	Etoposide	DGIdb	
**TP53**	Etoposide	DGIdb	
**GMNN**	Etoposide	DGIdb	
**CYP3A4**	Etoposide	DGIdb	
**GSTT1**	Etoposide	DGIdb	
**GDF15**	Etoposide	DGIdb	
**SOX2**	Etoposide		CTD
**MPO**	Etoposide		CTD
**MYC**	Etoposide		CTD
**COL4A1**	Etoposide		CTD
**CDKN1A**	Etoposide		CTD
**CDKN1B**	Etoposide		CTD
**EPHX1**	Etoposide		CTD
**RAD52**	Etoposide		CTD
**CDKN2A**	Etoposide		CTD
**FOXO3**	Etoposide		CTD
**CYP1A2**	Etoposide		CTD
**NRG1**	Etoposide		CTD
**DDR1**	Etoposide		CTD
**NPPA**	Etoposide		CTD
**ERBB2**	Etoposide		CTD
**ERBB3**	Etoposide		CTD
**PLK2**	Etoposide		CTD
**ERBB4**	Etoposide		CTD
**TRAF2**	Etoposide		CTD
**HIF1A**	Etoposide		CTD
**ATR**	Etoposide		CTD
**RB1**	Etoposide		CTD
**SPP1**	Etoposide		CTD
**FBL**	Etoposide		CTD
**AXL**	Etoposide		CTD
**FOS**	Etoposide		CTD
**GPX1**	Etoposide		CTD
**APAF1**	Etoposide		CTD
**GPX3**	Etoposide		CTD
**WT1**	Etoposide		CTD
**BAK1**	Etoposide		CTD

**CASP3**	Etoposide	CTD
**BAX**	Etoposide	CTD
**APEX1**	Etoposide	CTD
**BIRC2**	Etoposide	CTD
**LAMB2**	Etoposide	CTD
**CASP8**	Etoposide	CTD
**MCL1**	Etoposide	CTD
**BIRC3**	Etoposide	CTD
**CASP9**	Etoposide	CTD
**BIRC5**	Etoposide	CTD
**DAPK1**	Etoposide	CTD
**E2F1**	Etoposide	CTD
**CAT**	Etoposide	CTD
**RELA**	Etoposide	CTD
**CCND1**	Etoposide	CTD
**BCL2**	Etoposide	CTD
**BCL2L1**	Etoposide	CTD
**KEAP1**	Etoposide	CTD
**HMOX1**	Etoposide	CTD
**ITGA2B**	Etoposide	CTD
**PTEN**	Etoposide	CTD
**RUNX3**	Etoposide	CTD
**XRCC5**	Etoposide	CTD
**MDM2**	Etoposide	CTD
**FAS**	Etoposide	CTD
**FASLG**	Etoposide	CTD
**OGG1**	Etoposide	CTD
**GADD45A**	Etoposide	CTD
**PTGS2**	Etoposide	CTD
**RXRA**	Etoposide	CTD
**PHGDH**	Etoposide	CTD
**LGALS1**	Etoposide	CTD
**AREG**	Etoposide	CTD
**CEACAM1**	Etoposide	CTD
**ABCB1**	Etoposide	CTD
**CHUK**	Etoposide	CTD
**TFRC**	Etoposide	CTD
**TGFA**	Etoposide	CTD
**GSTM1**	Etoposide	CTD
**GSTM2**	Etoposide	CTD
**TYMS**	Etoposide	CTD
**CCNG1**	Etoposide	CTD
**GSTP1**	Etoposide	CTD
**GJA1**	Etoposide	CTD
**TYRP1**	Etoposide	CTD
**SLC22A18**	Etoposide	CTD

**MSH6**	Etoposide	CTD
**JUN**	Etoposide	CTD
**JUNB**	Etoposide	CTD
**NEK2**	Etoposide	CTD
**FASN**	Etoposide	CTD
**MT3**	Etoposide	CTD
**IGF1R**	Etoposide	CTD
**EGF**	Etoposide	CTD
**EGFR**	Etoposide	CTD
**EGR1**	Etoposide	CTD
**GCLC**	Etoposide	CTD
**MTOR**	Etoposide	CTD
**NFE2L2**	Etoposide	CTD
**KLF5**	Etoposide	CTD
**UCHL1**	Etoposide	CTD
**RASSF1**	Etoposide	CTD
**NFKB1**	Etoposide	CTD
**NFKBIA**	Etoposide	CTD
**PRDX1**	Etoposide	CTD
**CYCS**	Etoposide	CTD
**CHEK2**	Etoposide	CTD
**NQO1**	Etoposide	CTD
**CD274**	Etoposide	CTD
**KMT2A**	Etoposide	CTD
**ROBO1**	Etoposide	CTD
**SLC7A5**	Etoposide	CTD
**AKT1**	Etoposide	CTD
**HES1**	Etoposide	CTD
**CCN2**	Etoposide	CTD
**AVPI1**	Etoposide	CTD
**ATF3**	Etoposide	CTD
**TNF**	Etoposide	CTD
**PRKDC**	Etoposide	CTD
**MMP1**	Etoposide	CTD
**ATM**	Etoposide	CTD
**MAPK1**	Etoposide	CTD
**CTNNB1**	Etoposide	CTD
**MAPK3**	Etoposide	CTD
**IKBKB**	Etoposide	CTD
**IL1B**	Etoposide	CTD
**PLK1**	Etoposide	CTD
**FYN**	Etoposide	CTD
**KIT**	Etoposide	CTD
**ENO1**	Etoposide	CTD
**NOS2**	Etoposide	CTD
**IL6**	Etoposide	CTD

**EP300**	Etoposide	CTD
**TOP2A**	Etoposide	CTD
**WNK3**	Etoposide	CTD
**XRCC6**	Etoposide	CTD
**TP53**	Etoposide	CTD
**TP53BP1**	Etoposide	CTD
**SOD2**	Etoposide	CTD
**CXCL8**	Etoposide	CTD
**TP73**	Etoposide	CTD
**CDK2**	Etoposide	CTD
**SFN**	Etoposide	CTD
**VEGFA**	Etoposide	CTD
**TPI1**	Etoposide	CTD

Table S5. Target genes prediction of lung cancer

Gene name	Disease name	Data Source
**BCC1**	Lung cancer	OMIM
**CMM**	Lung cancer	OMIM
**MLM**	Lung cancer	OMIM
**DNS**	Lung cancer	OMIM
**DEL1p36**	Lung cancer	OMIM
**C1DELp36**	Lung cancer	OMIM
**SAI1**	Lung cancer	OMIM
**MTS1**	Lung cancer	OMIM
**TFS1**	Lung cancer	OMIM
**ISG15**	Lung cancer	OMIM
**G1P2**	Lung cancer	OMIM
**IFI15**	Lung cancer	OMIM
**IMD38**	Lung cancer	OMIM
**AGRN**	Lung cancer	OMIM
**CMS8**	Lung cancer	OMIM
**MIR200B**	Lung cancer	OMIM
**MIRN200B**	Lung cancer	OMIM
**MIR200A**	Lung cancer	OMIM
**MIRN200A**	Lung cancer	OMIM
**MIR429**	Lung cancer	OMIM
**MIRN429**	Lung cancer	OMIM
**TNFRSF4**	Lung cancer	OMIM
**TXGP1L**	Lung cancer	OMIM
**OX40**	Lung cancer	OMIM
**ACT35**	Lung cancer	OMIM
**IMD16**	Lung cancer	OMIM
**B3GALT6**	Lung cancer	OMIM
**SEMDJL1**	Lung cancer	OMIM
**EDSSPD2**	Lung cancer	OMIM
**ALGAZ**	Lung cancer	OMIM
**TAS1R3**	Lung cancer	OMIM

**T1R3**	Lung cancer	OMIM
**DVL1**	Lung cancer	OMIM
**DRS2**	Lung cancer	OMIM
**ATAD3C**	Lung cancer	OMIM
**ATAD3B**	Lung cancer	OMIM
**TOB3**	Lung cancer	OMIM
**KIAA1273**	Lung cancer	OMIM
**ATAD3A**	Lung cancer	OMIM
**HAYOS**	Lung cancer	OMIM
**PHRINL**	Lung cancer	OMIM
**ATAD3A**	Lung cancer	OMIM
**HAYOS**	Lung cancer	OMIM
**PHRINL**	Lung cancer	OMIM
**TMEM240**	Lung cancer	OMIM
**C1orf70**	Lung cancer	OMIM
**SCA21**	Lung cancer	OMIM
**MIB2**	Lung cancer	OMIM
**NADK**	Lung cancer	OMIM
**GNB1**	Lung cancer	OMIM
**MRD42**	Lung cancer	OMIM
**MDS**	Lung cancer	OMIM
**CFAP74**	Lung cancer	OMIM
**KIAA1751**	Lung cancer	OMIM
**C1orf222**	Lung cancer	OMIM
**CILD49**	Lung cancer	OMIM
**PRKCZ**	Lung cancer	OMIM
**PKC2**	Lung cancer	OMIM
**SKI**	Lung cancer	OMIM
**SGS**	Lung cancer	OMIM
**PLCH2**	Lung cancer	OMIM
**PLCL4**	Lung cancer	OMIM
**TNFRSF14**	Lung cancer	OMIM
**HVEM**	Lung cancer	OMIM
**TR2**	Lung cancer	OMIM
**PRDM16**	Lung cancer	OMIM
**MEL1**	Lung cancer	OMIM
**LVNC8**	Lung cancer	OMIM
**CMD1LL**	Lung cancer	OMIM
**PRDM16**	Lung cancer	OMIM
**MEL1**	Lung cancer	OMIM
**LVNC8**	Lung cancer	OMIM
**CMD1LL**	Lung cancer	OMIM
**ARHGEF16**	Lung cancer	OMIM
**MEGF6**	Lung cancer	OMIM
**EGFL3**	Lung cancer	OMIM
**MIR551A**	Lung cancer	OMIM

**WDR8**	Lung cancer	OMIM
**TP73**	Lung cancer	OMIM
**CILD47**	Lung cancer	OMIM
**SMIM1**	Lung cancer	OMIM
**VEL**	Lung cancer	OMIM
**LRRC47**	Lung cancer	OMIM
**KIAA1185**	Lung cancer	OMIM
**NPHP4**	Lung cancer	OMIM
**SLSN4**	Lung cancer	OMIM
**NPHP4**	Lung cancer	OMIM
**SLSN4**	Lung cancer	OMIM
**CHD5**	Lung cancer	OMIM
**PMNDS**	Lung cancer	OMIM
**HES2**	Lung cancer	OMIM
**TNFRSF25**	Lung cancer	OMIM
**TNFRSF12**	Lung cancer	OMIM
**DR3**	Lung cancer	OMIM
**LARD**	Lung cancer	OMIM
**PLEKHG5**	Lung cancer	OMIM
**KIAA0720**	Lung cancer	OMIM
**HMNR4**	Lung cancer	OMIM
**CMTRIC**	Lung cancer	OMIM
**PHF13**	Lung cancer	OMIM
**SPOC1**	Lung cancer	OMIM
**TNFRSF9**	Lung cancer	OMIM
**ILA**	Lung cancer	OMIM
**CD137**	Lung cancer	OMIM
**IMD109**	Lung cancer	OMIM
**DJ1**	Lung cancer	OMIM
**PARK7**	Lung cancer	OMIM
**MIG6**	Lung cancer	OMIM
**RALT**	Lung cancer	OMIM
**SLC45A1**	Lung cancer	OMIM
**DNB5**	Lung cancer	OMIM
**IDDNPF**	Lung cancer	OMIM
**ENO1**	Lung cancer	OMIM
**PPH**	Lung cancer	OMIM
**MPB1**	Lung cancer	OMIM
**MIR34A**	Lung cancer	OMIM
**MIRN34A**	Lung cancer	OMIM
**SLC25A33**	Lung cancer	OMIM
**PIK3CD**	Lung cancer	OMIM
**APDS**	Lung cancer	OMIM
**IMD14A**	Lung cancer	OMIM
**IMD14B**	Lung cancer	OMIM
**ROCHIS**	Lung cancer	OMIM

**CLSTN1**	Lung cancer	OMIM
**KIAA0911**	Lung cancer	OMIM
**CTNNBIP1**	Lung cancer	OMIM
**ICAT**	Lung cancer	OMIM
**LZIC**	Lung cancer	OMIM
**RBP7**	Lung cancer	OMIM
**CRBP4**	Lung cancer	OMIM
**KIF1B**	Lung cancer	OMIM
**CMT2A**	Lung cancer	OMIM
**CMT2A1**	Lung cancer	OMIM
**NBLST1**	Lung cancer	OMIM
**CENPS**	Lung cancer	OMIM
**APITD1**	Lung cancer	OMIM
**MHF1**	Lung cancer	OMIM
**DFFA**	Lung cancer	OMIM
**DFF1**	Lung cancer	OMIM
**CASZ1**	Lung cancer	OMIM
**SRG**	Lung cancer	OMIM
**C1orf127**	Lung cancer	OMIM
**CIROZ**	Lung cancer	OMIM
**HTX14**	Lung cancer	OMIM
**TARDBP**	Lung cancer	OMIM
**TDP43**	Lung cancer	OMIM
**ALS10**	Lung cancer	OMIM
**MASP2**	Lung cancer	OMIM
**MTOR**	Lung cancer	OMIM
**FRAP1**	Lung cancer	OMIM
**SKS**	Lung cancer	OMIM
**MTOR**	Lung cancer	OMIM
**FRAP1**	Lung cancer	OMIM
**SKS**	Lung cancer	OMIM
**DISP3**	Lung cancer	OMIM
**PTCHD2**	Lung cancer	OMIM
**KIAA1337**	Lung cancer	OMIM
**MAD2L2**	Lung cancer	OMIM
**MAD2B**	Lung cancer	OMIM
**FANCV**	Lung cancer	OMIM
**MTHFR**	Lung cancer	OMIM
**PLOD1**	Lung cancer	OMIM
**LH1**	Lung cancer	OMIM
**EDSKCL1**	Lung cancer	OMIM
**MFN2**	Lung cancer	OMIM
**KIAA0214**	Lung cancer	OMIM
**CMT2A2A**	Lung cancer	OMIM
**HMSN6A**	Lung cancer	OMIM
**CMT2A2B**	Lung cancer	OMIM

**MSL**	Lung cancer	OMIM
**TNFRSF8**	Lung cancer	OMIM
**CD30**	Lung cancer	OMIM
**D1S166E**	Lung cancer	OMIM
**TNFRSF1B**	Lung cancer	OMIM
**TNFR2**	Lung cancer	OMIM
**TNFBR**	Lung cancer	OMIM
**DHRS3**	Lung cancer	OMIM
**RETSDR1**	Lung cancer	OMIM
**PDPN**	Lung cancer	OMIM
**TI1A**	Lung cancer	OMIM
**T1A2**	Lung cancer	OMIM
**GP36**	Lung cancer	OMIM
**OTS8**	Lung cancer	OMIM
**AGGRUS**	Lung cancer	OMIM
**PRDM2**	Lung cancer	OMIM
**RIZ**	Lung cancer	OMIM
**FHAD1**	Lung cancer	OMIM
**EFHD2**	Lung cancer	OMIM
**SWS1**	Lung cancer	OMIM
**CTRC**	Lung cancer	OMIM
**CLCR**	Lung cancer	OMIM
**FBLIM1**	Lung cancer	OMIM
**FBLP1**	Lung cancer	OMIM
**MIGFILIN**	Lung cancer	OMIM
**SPEN**	Lung cancer	OMIM
**MINT**	Lung cancer	OMIM
**SHARP**	Lung cancer	OMIM
**HIAA0929**	Lung cancer	OMIM
**RATARS**	Lung cancer	OMIM
**SRARP**	Lung cancer	OMIM
**ERRF**	Lung cancer	OMIM
**C1orf64**	Lung cancer	OMIM
**HSPB7**	Lung cancer	OMIM
**CVHSP**	Lung cancer	OMIM
**EPHA2**	Lung cancer	OMIM
**ECK**	Lung cancer	OMIM
**CTPP1**	Lung cancer	OMIM
**CTPA**	Lung cancer	OMIM
**ARCC2**	Lung cancer	OMIM
**CTRCT6**	Lung cancer	OMIM
**FBXO42**	Lung cancer	OMIM
**FBX42**	Lung cancer	OMIM
**KIAA1332**	Lung cancer	OMIM
**SZRD1**	Lung cancer	OMIM
**C1orf144**	Lung cancer	OMIM

**RNU1A**	Lung cancer	OMIM
**RNU1**	Lung cancer	OMIM
**MFAP2**	Lung cancer	OMIM
**MAGP**	Lung cancer	OMIM
**MAGP1**	Lung cancer	OMIM
**ATP13A2**	Lung cancer	OMIM
**PARK9**	Lung cancer	OMIM
**KRPPD**	Lung cancer	OMIM
**SPG78**	Lung cancer	OMIM
**ATP13A2**	Lung cancer	OMIM
**PARK9**	Lung cancer	OMIM
**KRPPD**	Lung cancer	OMIM
**SPG78**	Lung cancer	OMIM
**SDHB**	Lung cancer	OMIM
**SDH2**	Lung cancer	OMIM
**SDHIP**	Lung cancer	OMIM
**PPGL4**	Lung cancer	OMIM
**MC2DN4**	Lung cancer	OMIM
**PADI1**	Lung cancer	OMIM
**PADI6**	Lung cancer	OMIM
**OZEMA16**	Lung cancer	OMIM
**PAX7**	Lung cancer	OMIM
**RMS2**	Lung cancer	OMIM
**CMYO19**	Lung cancer	OMIM
**UBR4**	Lung cancer	OMIM
**ZUBR1**	Lung cancer	OMIM
**RBAF600**	Lung cancer	OMIM
**KIAA1307**	Lung cancer	OMIM
**AKR7A3**	Lung cancer	OMIM
**AFAR2**	Lung cancer	OMIM
**D1S1733E**	Lung cancer	OMIM
**DAN**	Lung cancer	OMIM
**PLA2G2E**	Lung cancer	OMIM
**PLA2G2A**	Lung cancer	OMIM
**PLA2B**	Lung cancer	OMIM
**PLA2L**	Lung cancer	OMIM
**MOM1**	Lung cancer	OMIM
**PLA2G5**	Lung cancer	OMIM
**FRFB**	Lung cancer	OMIM
**PLA2G2F**	Lung cancer	OMIM
**MUL1**	Lung cancer	OMIM
**MULAN**	Lung cancer	OMIM
**C1orf166**	Lung cancer	OMIM
**CDA**	Lung cancer	OMIM
**PINK1**	Lung cancer	OMIM
**PARK6**	Lung cancer	OMIM

**EIF4G3**	Lung cancer	OMIM
**EIF4GII**	Lung cancer	OMIM
**ECE1**	Lung cancer	OMIM
**ECE1**	Lung cancer	OMIM
**ALPL**	Lung cancer	OMIM
**HOPS**	Lung cancer	OMIM
**TNSALP**	Lung cancer	OMIM
**HPPA**	Lung cancer	OMIM
**HPPO**	Lung cancer	OMIM
**HPPI**	Lung cancer	OMIM
**HPPC**	Lung cancer	OMIM
**USP48**	Lung cancer	OMIM
**USP31**	Lung cancer	OMIM
**DFNA85**	Lung cancer	OMIM
**HSPG2**	Lung cancer	OMIM
**PLC**	Lung cancer	OMIM
**SJS**	Lung cancer	OMIM
**SJA**	Lung cancer	OMIM
**SJS1**	Lung cancer	OMIM
**CDC42**	Lung cancer	OMIM
**TKS**	Lung cancer	OMIM
**WNT4**	Lung cancer	OMIM
**SERKAL**	Lung cancer	OMIM
**WNT4**	Lung cancer	OMIM
**SERKAL**	Lung cancer	OMIM
**EPHA8**	Lung cancer	OMIM
**EEK**	Lung cancer	OMIM
**HEK3**	Lung cancer	OMIM
**C1QA**	Lung cancer	OMIM
**C1QD1**	Lung cancer	OMIM
**EPHB2**	Lung cancer	OMIM
**EPHT3**	Lung cancer	OMIM
**DRT**	Lung cancer	OMIM
**ERK**	Lung cancer	OMIM
**PCBC**	Lung cancer	OMIM
**CAPB**	Lung cancer	OMIM
**BDPLT22**	Lung cancer	OMIM
**KDM1A**	Lung cancer	OMIM
**LSD1**	Lung cancer	OMIM
**AOF2**	Lung cancer	OMIM
**BHC110**	Lung cancer	OMIM
**KIAA0601**	Lung cancer	OMIM
**CPRF**	Lung cancer	OMIM
**AIMAH3**	Lung cancer	OMIM
**LUZP1**	Lung cancer	OMIM
**ASAP3**	Lung cancer	OMIM

**UPLC1**	Lung cancer	OMIM
**CENTB6**	Lung cancer	OMIM
**DDEFL1**	Lung cancer	OMIM
**ACAP4**	Lung cancer	OMIM
**E2F2**	Lung cancer	OMIM
**ID3**	Lung cancer	OMIM
**MDS2**	Lung cancer	OMIM
**RPL11**	Lung cancer	OMIM
**DBA7**	Lung cancer	OMIM
**CNR2**	Lung cancer	OMIM
**CB2**	Lung cancer	OMIM
**CX5**	Lung cancer	OMIM
**IFNLR1**	Lung cancer	OMIM
**IL28RA**	Lung cancer	OMIM
**STPG1**	Lung cancer	OMIM
**MAPO2**	Lung cancer	OMIM
**C1orf201**	Lung cancer	OMIM
**NIPAL3**	Lung cancer	OMIM
**NPAL3**	Lung cancer	OMIM
**RCAN3**	Lung cancer	OMIM
**DSCR1L2**	Lung cancer	OMIM
**MCIP3**	Lung cancer	OMIM
**CLIC4**	Lung cancer	OMIM
**MTCLIC**	Lung cancer	OMIM
**RUNX3**	Lung cancer	OMIM
**CBFA3**	Lung cancer	OMIM
**PEBP2A3**	Lung cancer	OMIM
**AML2**	Lung cancer	OMIM
**RSRP1**	Lung cancer	OMIM
**C1orf63**	Lung cancer	OMIM
**RHD**	Lung cancer	OMIM
**HDFNRH**	Lung cancer	OMIM
**RHD**	Lung cancer	OMIM
**HDFNRH**	Lung cancer	OMIM
**TMEM57**	Lung cancer	OMIM
**FLJ10747**	Lung cancer	OMIM
**LDLRAP1**	Lung cancer	OMIM
**ARH**	Lung cancer	OMIM
**FHCB2**	Lung cancer	OMIM
**FHCB1**	Lung cancer	OMIM
**FHCL4**	Lung cancer	OMIM
**MAN1C1**	Lung cancer	OMIM
**SELENON**	Lung cancer	OMIM
**SEPN1**	Lung cancer	OMIM
**SELN**	Lung cancer	OMIM
**CMYO3**	Lung cancer	OMIM

**STMN1**	Lung cancer	OMIM
**LAP18**	Lung cancer	OMIM
**SMN**	Lung cancer	OMIM
**SLC30A2**	Lung cancer	OMIM
**ZNT2**	Lung cancer	OMIM
**TNZD**	Lung cancer	OMIM
**ZNF593**	Lung cancer	OMIM
**ZT86**	Lung cancer	OMIM
**CATSPER4**	Lung cancer	OMIM
**SH3BGRL3**	Lung cancer	OMIM
**UBXN11**	Lung cancer	OMIM
**SOC**	Lung cancer	OMIM
**LIN28A**	Lung cancer	OMIM
**ARID1A**	Lung cancer	OMIM
**C1orf4**	Lung cancer	OMIM
**B120**	Lung cancer	OMIM
**SMARCF1**	Lung cancer	OMIM
**MRD14**	Lung cancer	OMIM
**CSS2**	Lung cancer	OMIM
**SFN**	Lung cancer	OMIM
**NR0B2**	Lung cancer	OMIM
**SHP**	Lung cancer	OMIM
**TENT5B**	Lung cancer	OMIM
**FAM46**	Lung cancer	OMIM
**FCN3**	Lung cancer	OMIM
**HAKA1**	Lung cancer	OMIM
**GPR3**	Lung cancer	OMIM
**WASF2**	Lung cancer	OMIM
**WAVE2**	Lung cancer	OMIM
**SCAR2**	Lung cancer	OMIM
**EBVS1**	Lung cancer	OMIM
**STX12**	Lung cancer	OMIM
**STX13**	Lung cancer	OMIM
**THEMIS2**	Lung cancer	OMIM
**C1orf38**	Lung cancer	OMIM
**ICB1**	Lung cancer	OMIM
**XKR8**	Lung cancer	OMIM
**PTAFR**	Lung cancer	OMIM
**ATP1F1**	Lung cancer	OMIM
**IF1**	Lung cancer	OMIM
**SEST2**	Lung cancer	OMIM
**HI95**	Lung cancer	OMIM
**YTHDF2**	Lung cancer	OMIM
**MECR**	Lung cancer	OMIM
**NRBF1**	Lung cancer	OMIM
**DYTOABG**	Lung cancer	OMIM

**OPA16**	Lung cancer	OMIM
**LAPTM5**	Lung cancer	OMIM
**SDC3**	Lung cancer	OMIM
**SYND3**	Lung cancer	OMIM
**SDCN**	Lung cancer	OMIM
**PUM1**	Lung cancer	OMIM
**KIAA0099**	Lung cancer	OMIM
**SCA47**	Lung cancer	OMIM
**NEDMSF**	Lung cancer	OMIM
**ZCCHC17**	Lung cancer	OMIM
**PS1D**	Lung cancer	OMIM
**FABP3**	Lung cancer	OMIM
**SERINC2**	Lung cancer	OMIM
**TINAGL1**	Lung cancer	OMIM
**TINAGRP**	Lung cancer	OMIM
**PTP4A2**	Lung cancer	OMIM
**PTP4A**	Lung cancer	OMIM
**PRL2**	Lung cancer	OMIM
**HH13**	Lung cancer	OMIM
**KHDRBS1**	Lung cancer	OMIM
**SAM68**	Lung cancer	OMIM
**TXLNA**	Lung cancer	OMIM
**IL14**	Lung cancer	OMIM
**CCDC28B**	Lung cancer	OMIM
**MGC1203**	Lung cancer	OMIM
**LCK**	Lung cancer	OMIM
**IMD22**	Lung cancer	OMIM
**HDAC1**	Lung cancer	OMIM
**RPD3L1**	Lung cancer	OMIM
**MARCKSL1**	Lung cancer	OMIM
**MLP**	Lung cancer	OMIM
**MRP**	Lung cancer	OMIM
**SYNC1**	Lung cancer	OMIM
**YARS1**	Lung cancer	OMIM
**YARS**	Lung cancer	OMIM
**CMTDIC**	Lung cancer	OMIM
**TYRRS**	Lung cancer	OMIM
**YTS**	Lung cancer	OMIM
**YRS**	Lung cancer	OMIM
**IMNEPD2**	Lung cancer	OMIM
**S100PBP**	Lung cancer	OMIM
**S100PBPR**	Lung cancer	OMIM
**FNDC5**	Lung cancer	OMIM
**FRCP2**	Lung cancer	OMIM
**AK2**	Lung cancer	OMIM
**AZIN2**	Lung cancer	OMIM

**ODC1L**	Lung cancer	OMIM
**KIAA1945**	Lung cancer	OMIM
**TRIM62**	Lung cancer	OMIM
**DEAR1**	Lung cancer	OMIM
**CSMD2**	Lung cancer	OMIM
**KIAA1884**	Lung cancer	OMIM
**HMGB4**	Lung cancer	OMIM
**GJB3**	Lung cancer	OMIM
**CX31**	Lung cancer	OMIM
**DFNA2B**	Lung cancer	OMIM
**EKVP1**	Lung cancer	OMIM
**TFAP2E**	Lung cancer	OMIM
**TEKT2**	Lung cancer	OMIM
**TEKTB1**	Lung cancer	OMIM
**NOR1**	Lung cancer	OMIM
**CSF3R**	Lung cancer	OMIM
**GCSFR**	Lung cancer	OMIM
**SCN7**	Lung cancer	OMIM
**CSF3R**	Lung cancer	OMIM
**GCSFR**	Lung cancer	OMIM
**SCN7**	Lung cancer	OMIM
**ZC3H12A**	Lung cancer	OMIM
**MCPIP**	Lung cancer	OMIM
**MCPIP1**	Lung cancer	OMIM
**SNIP1**	Lung cancer	OMIM
**NEDHCS**	Lung cancer	OMIM
**RSPO1**	Lung cancer	OMIM
**FLJ40906**	Lung cancer	OMIM
**AIRIM**	Lung cancer	OMIM
**C1orf109**	Lung cancer	OMIM
**YRDC**	Lung cancer	OMIM
**IRIP**	Lung cancer	OMIM
**GAMOS10**	Lung cancer	OMIM
**SF3A3**	Lung cancer	OMIM
**SF3A60**	Lung cancer	OMIM
**SAP61**	Lung cancer	OMIM
**PRP9**	Lung cancer	OMIM
**FHL3**	Lung cancer	OMIM
**SLIM2**	Lung cancer	OMIM
**RRAGC**	Lung cancer	OMIM
**RAGC**	Lung cancer	OMIM
**LNGODS**	Lung cancer	OMIM
**MYCBP**	Lung cancer	OMIM
**MACF1**	Lung cancer	OMIM
**ACF7**	Lung cancer	OMIM
**KIAA1251**	Lung cancer	OMIM

**LIS9**	Lung cancer	OMIM
**PPIE**	Lung cancer	OMIM
**CYP33**	Lung cancer	OMIM
**TRIT1**	Lung cancer	OMIM
**IPT**	Lung cancer	OMIM
**COXPD35**	Lung cancer	OMIM
**MYCL**	Lung cancer	OMIM
**LMYC**	Lung cancer	OMIM
**MFSD2A**	Lung cancer	OMIM
**NEDMISBA**	Lung cancer	OMIM
**PPT1**	Lung cancer	OMIM
**CLN1**	Lung cancer	OMIM
**RLF**	Lung cancer	OMIM
**ZMPSTE24**	Lung cancer	OMIM
**FACE1**	Lung cancer	OMIM
**STE24**	Lung cancer	OMIM
**MADB**	Lung cancer	OMIM
**RSDM1**	Lung cancer	OMIM
**MIR30C1**	Lung cancer	OMIM
**CITED4**	Lung cancer	OMIM
**SCMH1**	Lung cancer	OMIM
**GUCA2B**	Lung cancer	OMIM
**UGN**	Lung cancer	OMIM
**PPCS**	Lung cancer	OMIM
**CMD2C**	Lung cancer	OMIM
**YBX1**	Lung cancer	OMIM
**NSEP1**	Lung cancer	OMIM
**YB1**	Lung cancer	OMIM
**DBPB**	Lung cancer	OMIM
**FKBP5**	Lung cancer	OMIM
**GLDC**	Lung cancer	OMIM
**GLDC**	Lung cancer	OMIM
**SLC6A4**	Lung cancer	OMIM
**METTL21A**	Lung cancer	OMIM
**NEDD4L**	Lung cancer	OMIM
**BMP5**	Lung cancer	OMIM
**SERPINE1**	Lung cancer	OMIM
**CREB1**	Lung cancer	OMIM
**GRK5**	Lung cancer	OMIM
**RPP30**	Lung cancer	OMIM
**EHMT2**	Lung cancer	OMIM
**CD9**	Lung cancer	OMIM
**IL11**	Lung cancer	OMIM
**KDM4A**	Lung cancer	OMIM
**TP53**	Lung cancer	OMIM
**MAPK1**	Lung cancer	OMIM

**TGFB1**	Lung cancer	OMIM
**FGFR1**	Lung cancer	OMIM
**XIAP**	Lung cancer	OMIM
**ABCC3**	Lung cancer	OMIM
**MAPK3**	Lung cancer	OMIM
**PGP**	Lung cancer	OMIM
**ATM**	Lung cancer	OMIM
**TOP2A**	Lung cancer	OMIM
**E2F1**	Lung cancer	OMIM
**BCL2**	Lung cancer	OMIM
**ABCC1**	Lung cancer	OMIM
**TBP**	Lung cancer	OMIM
**BAX**	Lung cancer	OMIM
**SOX2**	Lung cancer	OMIM
**MPO**	Lung cancer	OMIM
**MYC**	Lung cancer	OMIM
**COL4A1**	Lung cancer	OMIM
**CDKN1A**	Lung cancer	OMIM
**CDKN1B**	Lung cancer	OMIM
**EPHX1**	Lung cancer	OMIM
**RAD52**	Lung cancer	OMIM
**CDKN2A**	Lung cancer	OMIM
**FOXO3**	Lung cancer	OMIM
**CYP1A2**	Lung cancer	OMIM
**NRG1**	Lung cancer	OMIM
**DDR1**	Lung cancer	OMIM
**NPPA**	Lung cancer	OMIM
**ERBB2**	Lung cancer	OMIM
**ERBB3**	Lung cancer	OMIM
**PLK2**	Lung cancer	OMIM
**ERBB4**	Lung cancer	OMIM
**TRAF2**	Lung cancer	OMIM
**HIF1A**	Lung cancer	OMIM
**ATR**	Lung cancer	OMIM
**RB1**	Lung cancer	OMIM
**SPP1**	Lung cancer	OMIM
**FBL**	Lung cancer	OMIM
**AXL**	Lung cancer	OMIM
**FOS**	Lung cancer	OMIM
**GPX1**	Lung cancer	OMIM
**APAF1**	Lung cancer	OMIM
**GPX3**	Lung cancer	OMIM
**WT1**	Lung cancer	OMIM
**BAK1**	Lung cancer	OMIM
**CASP3**	Lung cancer	OMIM
**BAX**	Lung cancer	OMIM

**APEX1**	Lung cancer	OMIM
**BIRC2**	Lung cancer	OMIM
**LAMB2**	Lung cancer	OMIM
**CASP8**	Lung cancer	OMIM
**MCL1**	Lung cancer	OMIM
**BIRC3**	Lung cancer	OMIM
**CASP9**	Lung cancer	OMIM
**BIRC5**	Lung cancer	OMIM
**DAPK1**	Lung cancer	OMIM
**E2F1**	Lung cancer	OMIM
**CAT**	Lung cancer	OMIM
**RELA**	Lung cancer	OMIM
**CCND1**	Lung cancer	OMIM
**BCL2**	Lung cancer	OMIM
**BCL2L1**	Lung cancer	OMIM
**KEAP1**	Lung cancer	OMIM
**HMOX1**	Lung cancer	OMIM
**ITGA2B**	Lung cancer	OMIM
**PTEN**	Lung cancer	OMIM
**RUNX3**	Lung cancer	OMIM
**XRCC5**	Lung cancer	OMIM
**MDM2**	Lung cancer	OMIM
**FAS**	Lung cancer	OMIM
**FASLG**	Lung cancer	OMIM
**OGG1**	Lung cancer	OMIM
**GADD45A**	Lung cancer	OMIM
**PTGS2**	Lung cancer	OMIM
**RXRA**	Lung cancer	OMIM
**PHGDH**	Lung cancer	OMIM
**LGALS1**	Lung cancer	OMIM
**AREG**	Lung cancer	OMIM
**CEACAM1**	Lung cancer	OMIM
**ABCB1**	Lung cancer	OMIM
**CHUK**	Lung cancer	OMIM
**TFRC**	Lung cancer	OMIM
**TGFA**	Lung cancer	OMIM
**GSTM1**	Lung cancer	OMIM
**GSTM2**	Lung cancer	OMIM
**TYMS**	Lung cancer	OMIM
**CCNG1**	Lung cancer	OMIM
**GSTP1**	Lung cancer	OMIM
**GJA1**	Lung cancer	OMIM
**TYRP1**	Lung cancer	OMIM
**SLC22A18**	Lung cancer	OMIM
**MSH6**	Lung cancer	OMIM
**JUN**	Lung cancer	OMIM

**JUNB**	Lung cancer	OMIM
**NEK2**	Lung cancer	OMIM
**FASN**	Lung cancer	OMIM
**MT3**	Lung cancer	OMIM
**IGF1R**	Lung cancer	OMIM
**EGF**	Lung cancer	OMIM
**EGFR**	Lung cancer	OMIM
**EGR1**	Lung cancer	OMIM
**GCLC**	Lung cancer	OMIM
**MTOR**	Lung cancer	OMIM
**NFE2L2**	Lung cancer	OMIM
**KLF5**	Lung cancer	OMIM
**UCHL1**	Lung cancer	OMIM
**RASSF1**	Lung cancer	OMIM
**NFKB1**	Lung cancer	OMIM
**NFKBIA**	Lung cancer	OMIM
**PRDX1**	Lung cancer	OMIM
**CYCS**	Lung cancer	OMIM
**CHEK2**	Lung cancer	OMIM
**NQO1**	Lung cancer	OMIM
**CD274**	Lung cancer	OMIM
**KMT2A**	Lung cancer	OMIM
**ROBO1**	Lung cancer	OMIM
**SLC7A5**	Lung cancer	OMIM
**AKT1**	Lung cancer	OMIM
**HES1**	Lung cancer	OMIM
**CCN2**	Lung cancer	OMIM
**AVPI1**	Lung cancer	OMIM
**ATF3**	Lung cancer	OMIM
**TNF**	Lung cancer	OMIM
**PRKDC**	Lung cancer	OMIM
**MMP1**	Lung cancer	OMIM
**ATM**	Lung cancer	OMIM
**MAPK1**	Lung cancer	OMIM
**CTNNB1**	Lung cancer	OMIM
**MAPK3**	Lung cancer	OMIM
**IKBKB**	Lung cancer	OMIM
**IL1B**	Lung cancer	OMIM
**PLK1**	Lung cancer	OMIM
**FYN**	Lung cancer	OMIM
**KIT**	Lung cancer	OMIM
**ENO1**	Lung cancer	OMIM
**NOS2**	Lung cancer	OMIM
**IL6**	Lung cancer	OMIM
**EP300**	Lung cancer	OMIM
**TOP2A**	Lung cancer	OMIM

**WNK3**	Lung cancer	OMIM
**XRCC6**	Lung cancer	OMIM
**TP53**	Lung cancer	OMIM
**TP53BP1**	Lung cancer	OMIM
**SOD2**	Lung cancer	OMIM
**CXCL8**	Lung cancer	OMIM
**TP73**	Lung cancer	OMIM
**CDK2**	Lung cancer	OMIM
**SFN**	Lung cancer	OMIM
**VEGFA**	Lung cancer	OMIM
**TPI1**	Lung cancer	OMIM
